# Novel Peptide-Calix[4]arene Conjugate Inhibits Aβ Aggregation and Rescues Neurons from Aβ’s Oligomers Cytotoxicity *In Vitro*

**DOI:** 10.1021/acschemneuro.1c00117

**Published:** 2021-04-12

**Authors:** Grazia Maria Letizia Consoli, Rita Tosto, Ausilia Baglieri, Salvatore Petralia, Tiziana Campagna, Giuseppe Di Natale, Stefania Zimbone, Maria Laura Giuffrida, Giuseppe Pappalardo

**Affiliations:** † CNR-Institute of Biomolecular Chemistry, Via P. Gaifami 18, 95126 Catania, Italy; ‡ International PhD School of Chemical Sciences, University of Catania, V.le A. Doria 6, 95125 Catania, Italy; § CNR-Institute of Crystallography, Via P. Gaifami 18, 95126 Catania, Italy; ∥ Department of Drug Sciences and Health, University of Catania, V.le A. Doria 6, 95125 Catania, Italy

**Keywords:** Aβ oligomers, amyloid, calixarenes, peptides, SH-SY5Y cells

## Abstract

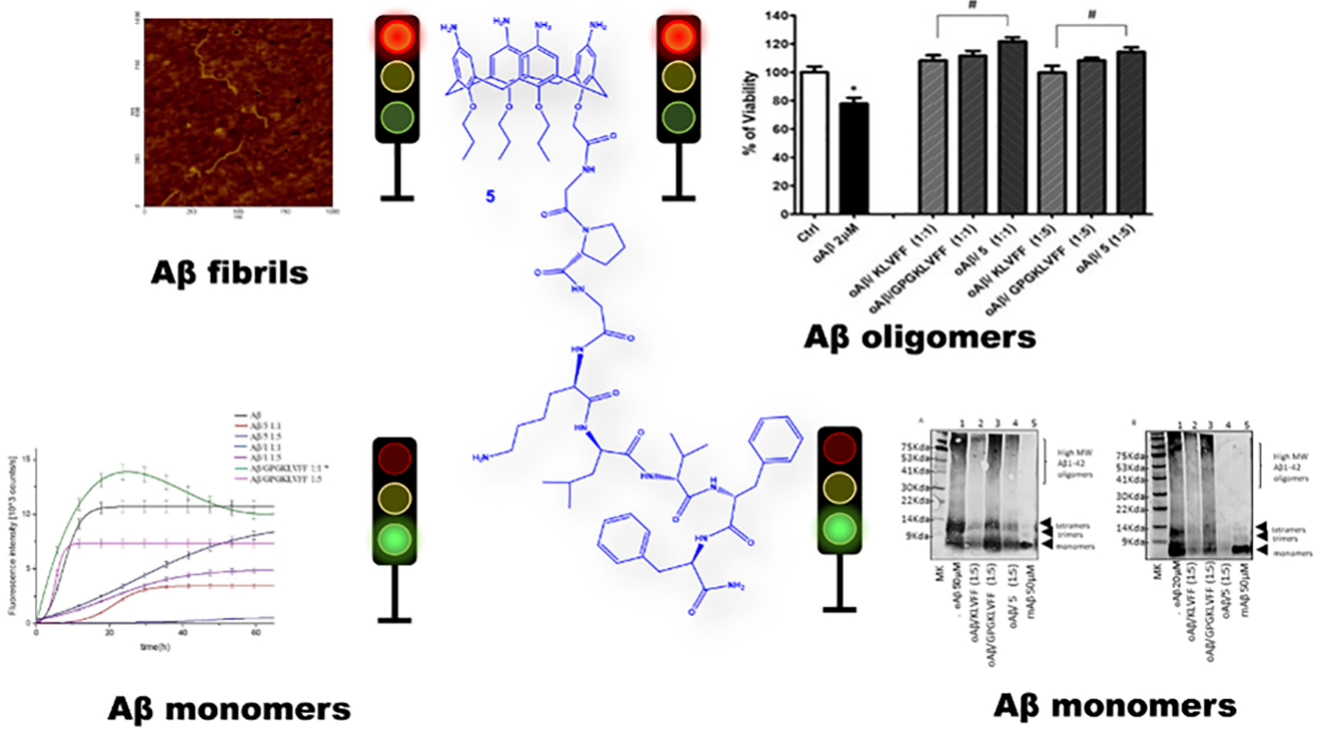

Alzheimer’s disease (AD) is a progressive neurodegenerative condition affecting people in the elderly. Targeting aggregation of β-amyloid peptides (Aβ) is considered a promising approach for the therapeutic treatment of the disease. Peptide based inhibitors of β-amyloid fibrillation are emerging as safe drug candidates as well as interesting compounds for early diagnosis of AD. Peptide conjugation via covalent bond with functional moieties enables the resultant hybrid system to acquire desired functions. Here we report the synthesis, the structural characterization, and the Aβ_42_ interaction of a *p*-amino-calix[4]arene derivative bearing a GPGKLVFF peptide pendant at the lower rim. We demonstrate that the *p*-amino-calix[4]arene–GPGKLVFF conjugate alters the Aβ_42_ aggregation pathways by preventing Aβ_42_’s conformational transition from random coil to β-sheet with concomitant changes of the aggregation kinetic profile as evidenced by circular dichroism (CD), thioflavin T (ThT), and dynamic light scattering (DLS) measurements, respectively. High resolution mass spectrometry (HR-MS) confirmed a direct interaction of the *p*-amino-calix[4]arene–GPGKLVFF conjugate with Aβ_42_ monomer which provided insight into a possible working mechanism, whereas the alteration of the Aβ_42_’s fibrillary architecture, by the calix-peptide conjugate, was further validated by atomic force microscopy (AFM) imaging. Finally, the herein proposed compound was shown to be effective against Aβ_42_ oligomers’ toxicity in differentiated neuroblastoma cells, SH-SY5Y.

## Introduction

Alzheimer’s disease (AD) is associated with a progressively neurodegenerative condition leading to dementia that mostly affects older people. AD represents one of the “protein misfolding diseases” with the highest socioeconomic impact^1^ and still denotes a serious threat and an important challenge for scientific research in both the therapeutic and diagnostic fields. The cause and progression of the disease are not yet fully elucidated, being AD, especially the sporadic, late onset form of the disease (LOAD), linked to genetic or environmental risk factors, many of which are not still completely clarified.^2^ Currently, the therapeutic treatments, available in the market, offer only small symptomatic benefits and in part can slow down the course of the disease. One of the pathological hallmarks of AD is the presence of extracellular plaques mainly composed of aggregated forms of amyloid-β (Aβ) peptide, a 39–43 residue fragment derived from the amyloid precursor protein (APP).^3^ Recent studies demonstrate that early stage prefibrillar aggregates such as oligomers and protofibrils are highly toxic species in the central nervous system compared to mature fibrils.^4,5^ Structural information about the toxic oligomeric Aβ species underlying AD has been difficult to obtain at an atomic level.^6^ The transient and heterogeneous properties of these assemblies imposes many challenges for the understanding of the multiple toxic mechanisms. Some oligomers are proposed to impart their toxic function by interacting directly with the cell membrane of neurons with consequent permeabilization and disruption.^7,8^ Thus, unveiling the structural features of oligomeric Aβ species is turning a topical field of investigation propaedeutic for the design of effective therapeutics that target these pathogenic Aβ species.^9,10^ Aβ_42_ aggregates into 6–10 nm diameter fibrils with the characteristic cross-β structure.^3^ Preventing Aβ_42_ peptide aggregation is therapeutically attractive, since Aβ aggregation is an exclusively pathological process.^11^ Selective targeting of Aβ’s fibrillogenesis should not interfere with the physiological function of APP as well as other proteins involved in the production of Aβ monomers, whose physiological beneficial role has been recently reported.^12^ Several small molecules, metal chelators, carbohydrate-containing compounds, and short peptides have been identified as inhibitors of amyloid aggregation.^13−16^ In particular, the clinical evidence of an abnormal metal ion interaction with Aβ in AD has promoted studies aimed at developing potential therapeutic strategies by using metal chelators or antioxidant compounds that target aberrant metal distribution and the adverse consequences of metal induced oxidative stress in AD.^17−19^ The finding that the hydrophobic core at residues 16–20 of Aβ (KLVFF) is crucial for the formation of β-sheet structures^20^ has stimulated the investigation of the KLVFF peptide as an inhibitor of Aβ_42_ fibrillogenesis.^21^ Some papers in the past reported that the KLVFF peptide, by binding the homologous sequence in full-length Aβ, can prevent at aggregation into fibrils^13,22,23^ and this ability is maintained after conjugation to different scaffolds including oligolysines,^24^ cyclodextrins,^25^ dendrimers,^26^ or porphyrins.^27,28^

Therefore, conjugation has emerged as a popular mechanism to modify or enhance the properties of a peptide drug candidate.^29,30^ Conjugation can also be used to deliver a cytotoxic payload or imaging agent to specific cell types targeted by the peptide.^31^ Calix[*n*]arenes are macrocyclic polyphenols proposed as molecular scaffolds for different fields of application.^32^ These macrocycles due to the peculiar structure and synthetic versatility have gained great interest in supramolecular chemistry. The calix[4–8]arene oligomers possess a hydrophobic cavity able to host a variety of guests and can cluster and spatially organize multiple ligands, providing rigid or flexible constructs suited for molecular recognition events. The low toxicity and immunogenicity exhibited by a variety of calix[*n*]arene derivatives have advanced these macrocycles to applications in biomedical and pharmaceutical fields.^33^ Calix[*n*]arene derivatives have been proposed as anticancer,^34^ antibacterial,^35^ synthetic vaccines,^36^ imaging agents,^37^ and drug^38^ and gene^39^ delivery systems. Opportunely functionalized, the calix[*n*]arenes have also been exploited as biomimetic models to better understand relevant biological processes underlying the protein–protein or protein–carbohydrate interactions.^40^ Yet, calix[*n*]arenes have been successfully used as novel compounds for protein detection^40^ and protein modulation.^41^ However, despite the wide use of calix[*n*]arenes in several fields of application, very few papers have dealt with the interactions of calix[*n*]arene derivatives with Aβ. Wang et al. described *p*-sulfonate-calix[4,6,8]arenes with anti- and disaggregating effect on Aβ_42_ through nonspecific hydrophobic interactions.^42^ Guo et al. synthesized a nanoassembly consisting of multiple units of a *p*-sulfonate calix[4]arene and a β-cyclodextrin, which by means of the multiple complexing cavities exposed on the nanoassembly surface bind Aβ_42_, inhibiting its aggregation or inducing fibril disaggregation.^43^ We determined that endowing the calixarene macrocycle with an Aβ recognition motif would generate a more refined tool to contrast the detrimental effects of the aggregated forms of Aβ peptides. Thus, we resorted to the design and synthesis of a new calix[4]arene–peptide conjugate composed of a *p*-amino-calix[4]arene derivative bearing a GPGKLVFF peptide sequence at the macrocycle lower rim. We describe herein the synthesis and structural characterization of this hybrid system, alongside its capability to hamper Aβ_42_ aggregation *in vitro*. The study has been carried out using a variety of spectroscopic techniques including circular dichroism (CD), thioflavin T (ThT) fluorescence, dynamic light scattering (DLS), and atomic force microscopy (AFM) imaging. High resolution mass spectrometry (HR-MS) was employed to point out direct interaction of the *p*-amino-calix[4]arene-GPGKLVFF conjugate with the Aβ_42_ monomer. We also aim at providing *in vitro* experimental evidence of the ability of this novel construct to prevent the Aβ oligomer cytotoxic effects on differentiated SH-SY5Y neuronal cultures. We demonstrate that the calix–peptide conjugate is *per se* nontoxic to neuronal cells and this allows its potential use as therapeutic agent in AD.

## Results and Discussion

### Design and Synthesis of Calix[4]arene–GPGKLVFF (**5**)

In search of functional calix[*n*]arene inhibitors of Aβ amyloid fibrillogenesis, we thought that joining an Aβ recognition peptide moiety to the framework of a water-soluble *p*-amino-calix[4]arene derivative would result in a new hybrid construct able to efficiently interact with Aβ peptides and therefore hampering peptide chain’s self-assembly into oligomeric/fibrillary toxic species. We chose the GPGKLVFF sequence because of the established ability of the KLVFF motif to recognize the homologous region of the parent full-length Aβ peptide. The additional GPG tripeptide was inserted to reduce any eventual propensity to self-aggregation of the calix–peptide conjugate. The *p*-amino-calix[4]arene scaffold should confer enhanced water solubility to the resultant calix–peptide conjugate, at the same time establishing multiple noncovalent contacts with the aromatic and anionic amino acid residues of the Aβ peptide. Moreover, it is expected that the calix–peptide conjugate would generate a steric hindrance between adjacent peptide chains thereby disturbing the Aβ’s self-assembly process. Compared to other peptide-conjugated systems, the peptide–calixarene difunctional compound can offer some advantages deriving from the known complexing properties of the calixarene cavity, in addition to the antiamyloidogenic action of β-sheet breaker pentapeptide KLVFF as a “binding element” for targeting Aβ. The calixarene moiety might also synergically assist the peptide ligand in both Aβ monomer stabilization and protection of neurons from Aβ oligomeric insult. The calix–peptide hybrid system might in principle be useful for delivery purposes of AD active compounds or imaging agents for theragnostic application.

**Scheme 1:**
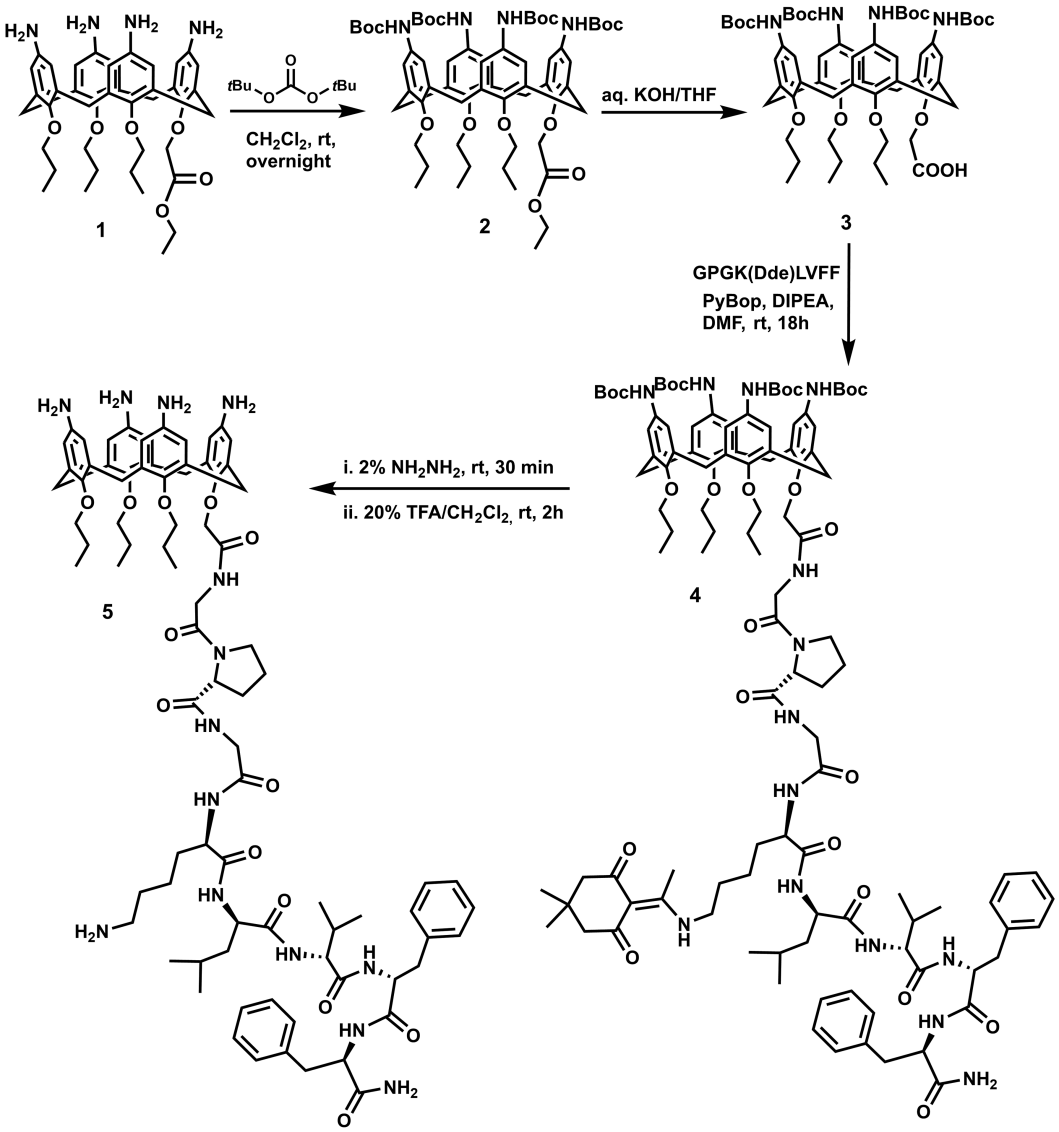
Synthetic Route for the Preparation of the *p*-Amino-calix[4]arene–GPGKLVFF Conjugate (**5**)

The procedure for the preparation of the calix-peptide conjugate (**5**) starting from compound **1**(45) is depicted in Scheme 1. In brief, for the preparation of compound **1**(45) the starting material *p-tert*-butyl-calix[4]arene was blocked in a cone conformation by tetra-functionalization of its lower rim with three propyl groups and one ethyl acetate group. Amino groups were introduced at the calix[4]arene upper rim, by ipso-nitration followed by C/Pd catalyzed reduction of the nitro groups. Before the coupling reaction with the peptide fragment, the amino groups at the upper rim were protected by *tert*-butyloxycarbonyl (Boc) groups to give compound **2**. Hydrolysis reaction provided compound **3** in which the COOH group at the lower rim can be used for the linkage of the GPGK(Dde)LVFF pendant via amide bond (Dde: N-(1-(4,4-dimethyl-2,6-dioxocyclohexylidene)ethyl). The GPGK(Dde)LVFF sequence was synthesized by using the standard microwave assisted Solid Phase Peptide Synthesis (mw-SPPS) reported in the literature,^46^ and characterized by HR-ESI-MS (see Figure S1 Supporting Information). Peptide conjugation to the calix[4]arene scaffold was accomplished in solution and in the presence of PyBop as a condensing agent. The selective removal of the Dde and Boc protecting groups, by sequential treatment with hydrazine and trifluoroacetic acid respectively, gave the compound **5** whose molecular identity was confirmed by combined MALDI-TOF-MS, HR-ESI-MS and 1*D*/2D NMR spectra (Supporting Information Figures S2–S10).

### Calix[4]arene–GPGKLVFF (**5**)/Aβ_42_ Interactions

To assess the inhibitory properties of **5** toward Aβ_42_ fibril formation, we carried out a combined CD and ThT fluorescence study. DLS and AFM measurements aided the determination of the size and morphology of the Aβ_42_ aggregates, respectively.

#### CD Spectroscopy

**Figure 1:**
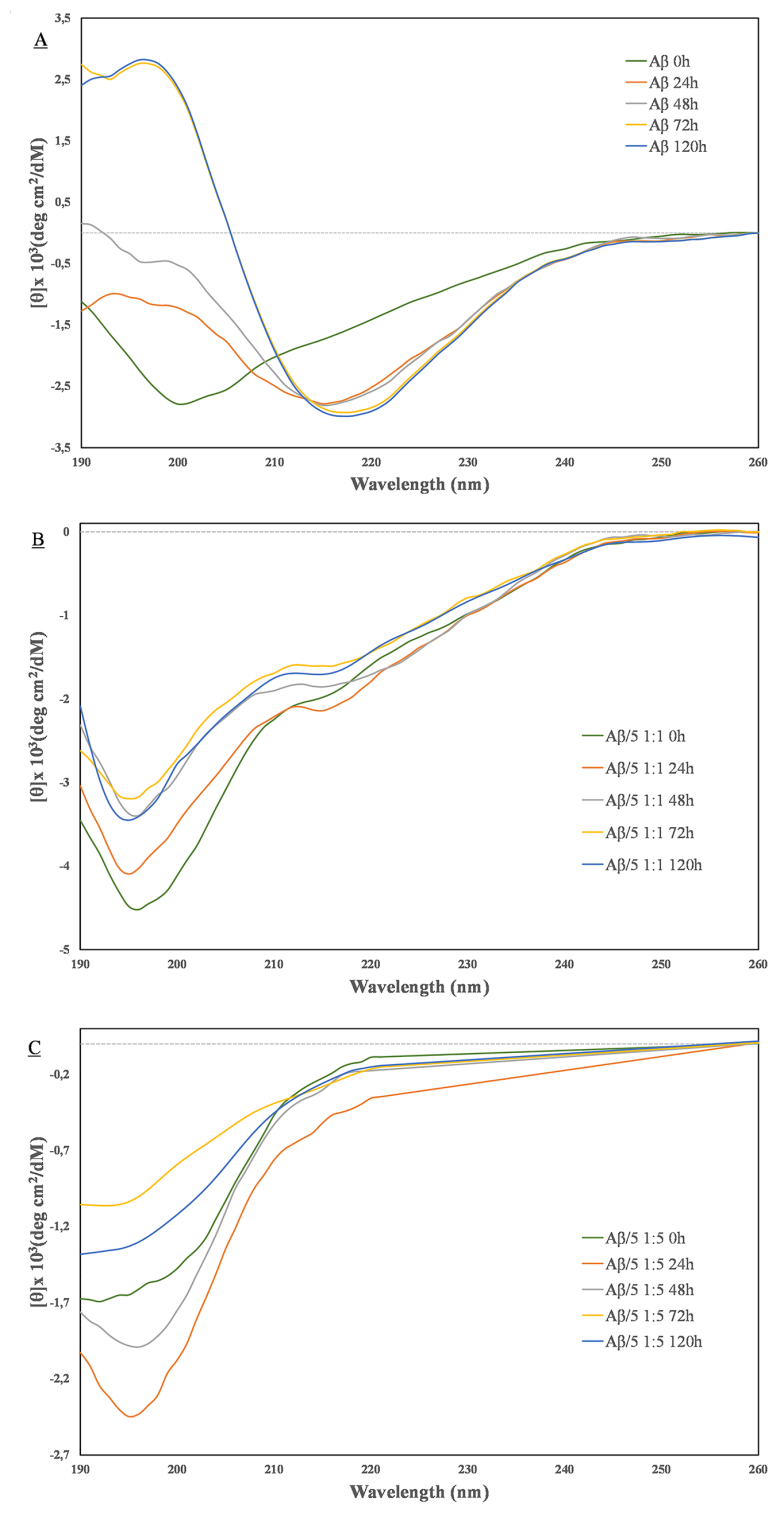
CD spectra of (A) Aβ_42_, (B) Aβ_42_/**5** (1:1 molar ratio), and (C) Aβ_42_/**5** (1:5 molar ratio) recorded at different times.

**Figure 2:**
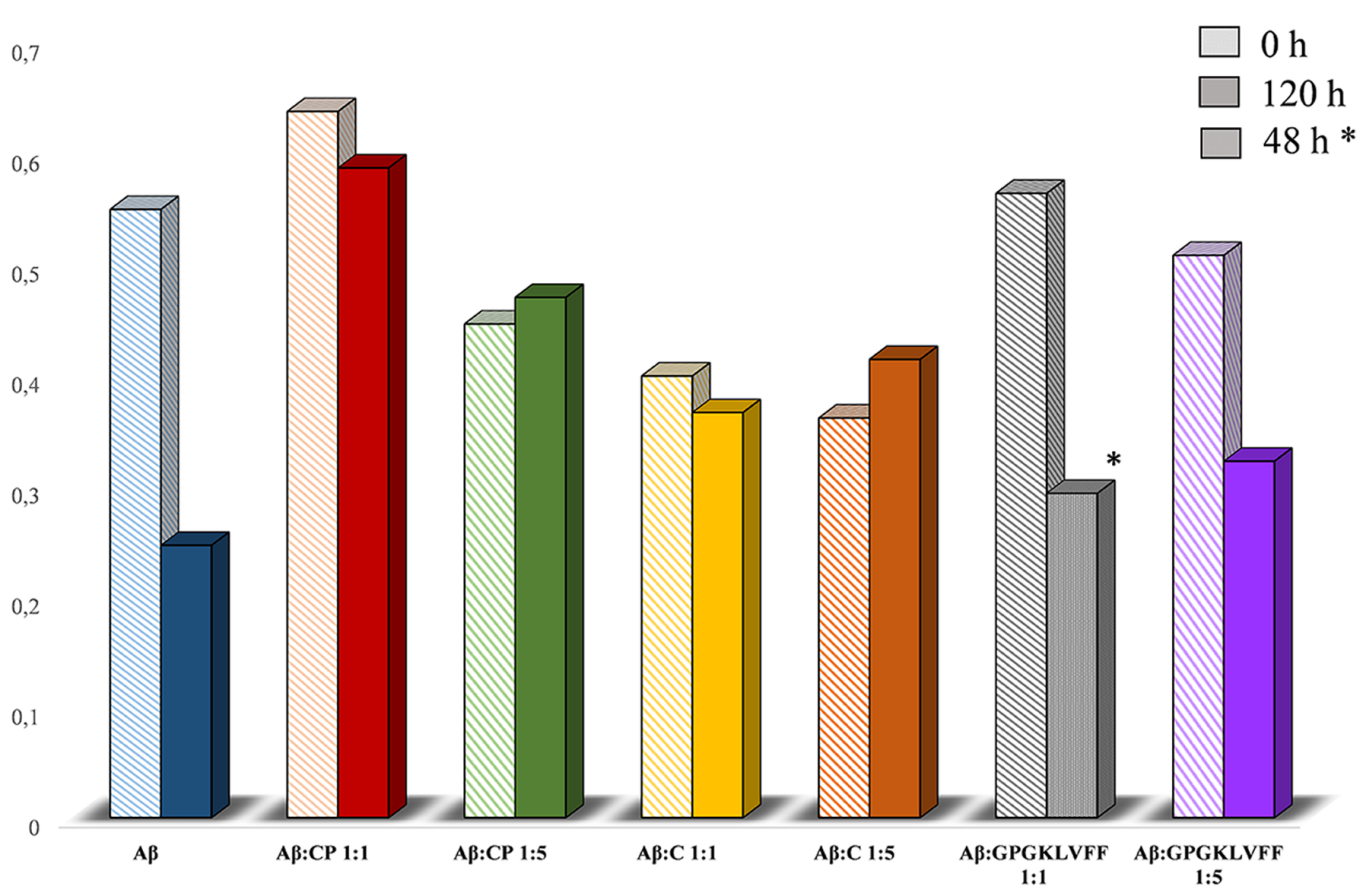
Graphical representation of the average percentage of unordered peptide conformation as determined by using CONTIN and CDSSTR deconvolution algorithms. CP = conjugate **5**; C = compound **1**. *Refers to 48 h incubation because of massive sample precipitation.

The conformational transition from a random coil to a β-sheet structure is the crucial step for the fibrillogenesis of Aβ_42_.^47^ A series of time course CD experiments, either with pure Aβ_42_ samples or Aβ_42_ coincubated with compounds **1** or **5** in phosphate buffer (10 mM, pH 7.4), were carried out to examine the effect of these derivatives on the Aβ_42_’s β-sheet conformational transition. Figure 1 shows the CD spectra recorded for Aβ_42_ alone and in the presence of compound **5**. It is clear from Figure 1 that, in the absence of **5**, the Aβ_42_ sample (5 μM) undergoes to an almost complete β-sheet conformational transition after 72 h. In fact, the freshly prepared Aβ_42_ sample displays clear negative dichroism below 200 nm that is typical of a randomly coiled peptide chain. This curve profile gradually changes toward the β-sheet pattern, as the incubation time proceeds. The CD spectrum recorded at 72 h incubation time shows positive ellipticity at 190 nm along with a negative signal at 218 nm, thereby indicating the presence of β-sheet peptide structures.^48^ This pattern does not change significantly after this time, indicating that Aβ_42_ almost reaches the final state after 5 days of incubation. The CD spectra recorded in the presence of **5**, either at equimolar or 5 mol excess, never display the characteristic β-sheet pattern. More interestingly, the observed CD spectra recorded at 5-fold molar excess always exhibit strong negative ellipticity at 190 nm along with no apparent inflection of the negative band around 218 nm in the considered interval of time.^48^ The experimental CD curves were subjected to deconvolution analysis using the CONTINN and CDSSTR algorithms.^49^ In Figure 2, the graphical representation of the percentage of unordered peptide conformation as estimated at *t* = 0 or *t* = 120 h is reported. The values of the determined random coil secondary structure are reported in Table S1. It is apparent that at *t* = 120 h a higher percentage of random coil conformation is maintained in the presence of the derivative **5** with respect to Aβ_42_ control. The CD spectra of the calix–peptide conjugate **5** alone were recorded in the same experimental conditions. These spectra are characterized by a generalized low CD amplitude that does not change with time (Figure S11). All the above indicates that **5** significantly inhibits the Aβ_42_’s β-sheet conformational transition typically associated with the fibrillogenesis process.^47^ In other words, the CD results suggest that **5** well preserves the Aβ randomly coiled monomer from its recruitment into potentially toxic aggregates. We wanted also to evaluate the ability of the calix[4]arene macrocycle to interfere with the random coil/β-sheet conformational transition of Aβ_42_. We then acquired a new set of CD measurements, under the same experimental conditions as above, on a sample of Aβ_42_ and the *p*-amino-calix[4]arene derivative **1** in both 1:1 and 1:5 molar ratios. CD data showed that compound **1** is able to interact with Aβ_42_ (Figure S12), although to a lesser extent than **5**. This also became evident from the deconvolution analysis (Figure 2) where the percentage of unordered peptide chain is higher than the one of the Aβ_42_ control at *t* = 120 h. The inhibitory effect of the unconjugated GPGKLVFF on Aβ’s self-assembly process was also evaluated for comparison. As expected, the CD data demonstrate the ability of the free peptide to interfere with the Aβ aggregation (Figure S13). However, the comparison with the effects generated by the synthesized conjugate **5** allows us to point out some differences. Unlike the case of compound **5**, at the 1:1 ratio with Aβ, the unconjugated GPGKLVFF peptide is able to accelerate the fibrillogenic process. This turns evident in the CD experiments (Figure S13). A typical CD profile of the β-sheet conformation was observed after 48 h coincubation (Aβ alone reaches the same conformation after 72 h). The acceleration of the aggregation rate caused massive precipitation, and we were not able to acquire CD spectra at 72 h (Figure S13). The CD data obtained at a 1:5 ratio indicated that the free GPGKLVFF can slow down the aggregation process of Aβ but, in any case, to a lesser extent than the one observed in the presence of the calix–peptide conjugate at both 1:1 and 1:5 ratios. In conclusion, the CD study suggests that compounds **1** and free GPGKLVFF can affect the Aβ_42_ propensity to adopt β-sheet conformation. Importantly, the CD experiments clearly indicated the superiority of the hybrid system **5** in inhibiting Aβ_42_ aggregation compared to the separate calix or peptide moieties.

#### Thioflavin T Fluorescence Assay (ThT)

**Figure 3:**
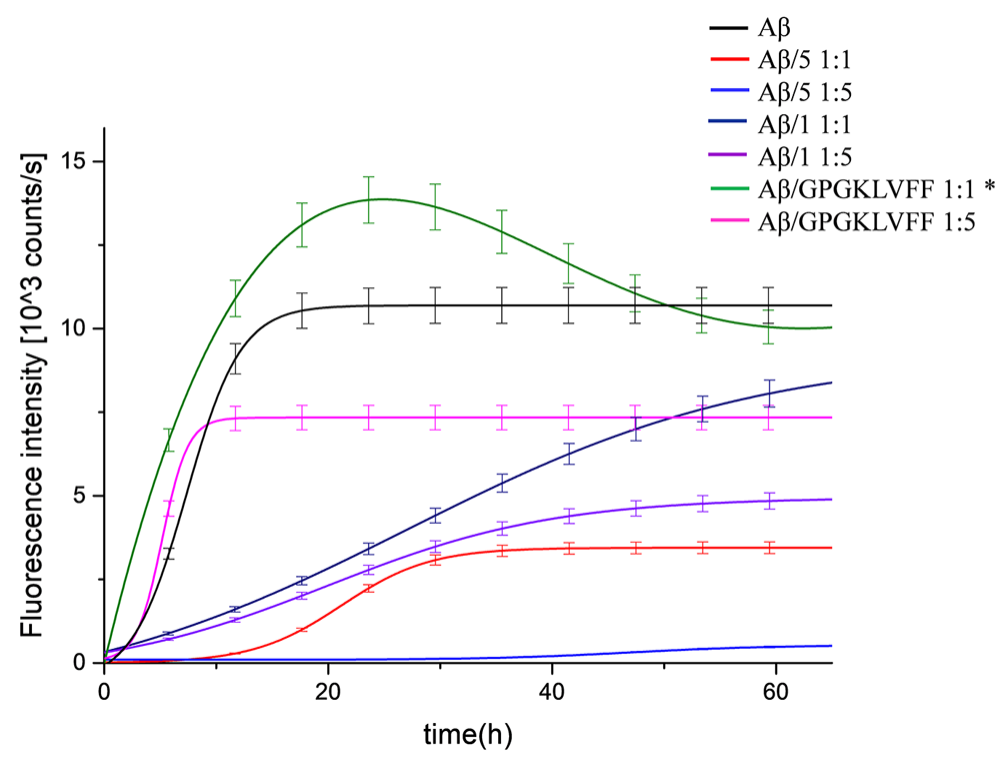
Aggregation kinetics detected by in situ ThT fluorescence assays of Aβ_42_ (20 μM, black) and in the presence of equimolar or 5-fold molar excess of compound **1**, **5** or GPGKLVFF (Aβ_42_/**1** 1:1, dark blue), (Aβ_42_/**1**1:5, purple), (Aβ_42_/**5** 1:1, red), (Aβ_42_/**5**, 1:5 light blue), (Aβ_42_/GPGKLVFF 1:1, green), (Aβ_42_/GPGKLVFF 1:5, pink). *The extraction of the kinetic parameters by using the empirical equation in the Experimental Section was not possible.

The kinetic of Aβ_42_ aggregation was also monitored by ThT fluorescence. ThT is a fluorophore that enhances its fluorescence intensity upon binding to amyloid fibrils.^50^ Fluorescence intensity was monitored at 482 nm for 67 h and at 37 °C. The results are presented in Figure 3. In the absence of inhibitors, Aβ_42_ displayed a rapid increase of the ThT fluorescence, as expected from the inherent propensity to amyloid formation of this peptide. It is also evident that the curve profile observed displays just a hint of initial nucleation phase (Figure 3, black). The prompt formation of aggregated seeds (at *t* = 0) might be the responsible of such behavior. It is assumed that the lower fluorescence intensity we observed in the presence of the inhibitors at either equimolar ratio or 5-fold molar excess is related to a lesser amount of Aβ_42_ fibrillary aggregated form. The antifibrillogenic activity of the conjugate peptide **5** is evident at equimolar ratio (Figure 3, red). Interestingly the observed profile displays a clear nucleation phase (lag phase), that suggests an effective action of compound **5** in the early events of Aβ_42_ fibrillogenesis.

Such an effect becomes even more apparent at 5-fold molar excess. As one can see, a very long lag phase, along with a negligible increasing of ThT fluorescence intensity, testifies an almost complete inhibition of fibril formation (Figure 3, light blue). The ThT curves related to the kinetic of the calixarene precursor **1** or free GPGKLVFF with Aβ_42_ also reveal the capability of these compounds to interfere with the Aβ_42_ fibrillogenic process, although to a lesser extent than compound **5**. What we observed was a reduction of the fluorescence intensity of the curves, but, again, a clear lag phase was not observed. The only exception was the ThT curve observed at 1:1 ratio for the free GPGKLVFF. It strongly activated Aβ fibril generation (Figure 3, green). This proaggregating behavior is in keeping with the CD measurements, and substantial flocculation appeared after 48 h. The sample reaches ThT fluorescence max intensity faster than Aβ_42_ alone. It might be conceivable that the compound may somehow self-aggregate and give false positive, but control ThT experiments ruled out significant interference occurring between ThT and compounds **1**, **5** or GPGKLVFF (Figure S14). An alternative interpretation implies the ability of free GPGKLVFF compound to become an integral part of ThT positive heterofibrils with Aβ^51^ (Figure 3, pink). The whole of the ThT results further confirm that both **1** and **5** exhibit a dose responsive antifibrillogenic activity. A quantitative analysis of the experimental curves was obtained by proper fitting using a suitable equation, and the relevant kinetic parameters are reported in the Supporting Information (Table S2).

#### Dynamic Light Scattering (DLS)

**Figure 4:**
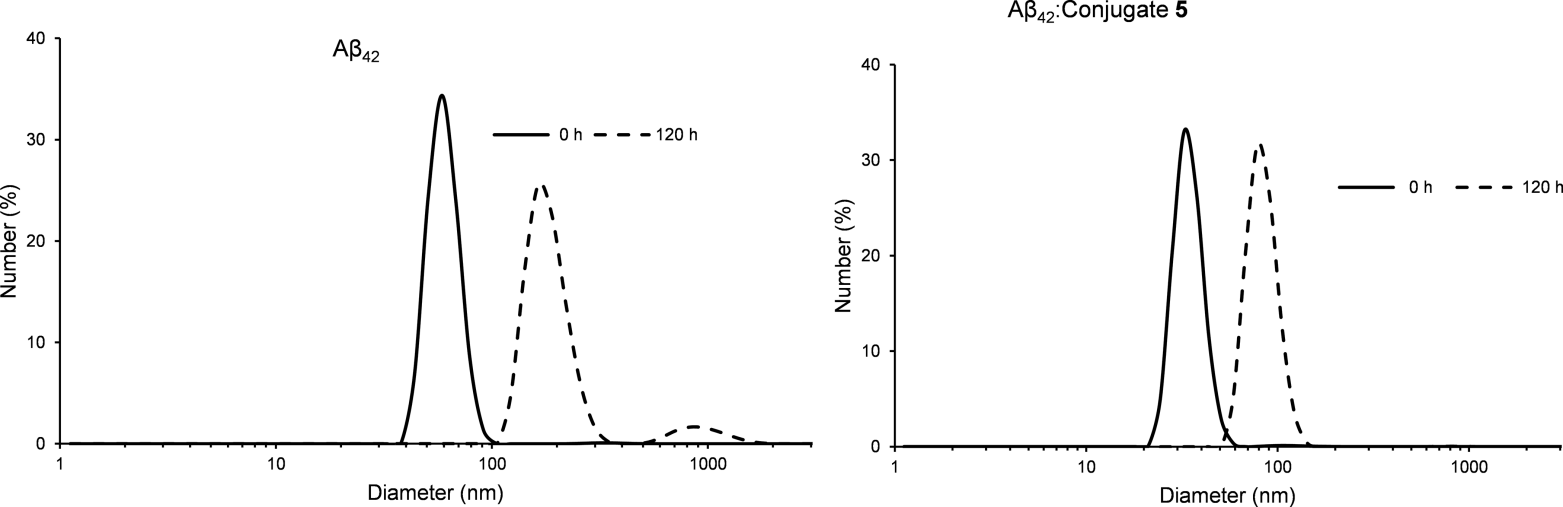
DLS size distributions by number (%) for Aβ_42_ (5 μM) and Aβ_42_ in the presence of conjugate **5** (Aβ_42_/**5** 1:1) at *t* = 0 and after 120 h incubation at 37 °C in 10 mM phosphate buffer.

We resorted to DLS measurements to determine the average size of the aggregates in solution and to monitor their growth over time, either in the absence or in the presence of **5**. Data were collected at *t* = 0 and 120 h and are shown in Figure 4 as the analysis of the number of scattering objects.^52^ The analysis indicates that small aggregates with an average size of 60 nm are the main scattering objects in the freshly prepared Aβ_42_ sample (*t* = 0). The end stage of Aβ aggregation was analyzed after 120 h, and the formation of larger aggregates was observed. In particular, the DLS profile indicated the presence of particles with increased diameter values (>100 nm) as the most abundant scattering entities, along with a very low percentage of bigger aggregates with size in the micrometric range. The data collected in the presence of **5** clearly indicated the reduction of the dimension of Aβ_42_ aggregates both at the initial stage and end point of monitoring. In fact, at *t* = 0, the majority of the scattering particles in solution displayed a diameter size of around 45 nm (Figure 4), whereas only aggregates as large as 84 nm were detected after 120 h. It should be said that compound **5** alone (5 μM) formed small aggregates with a mean hydrodynamic diameter around 8 nm at *t* = 0. Larger aggregates (diameter > 300 nm) were seen at *t* = 120 h (Figure S15). At 20 μM, compound **5** forms nanoaggregates with a mean hydrodynamic diameter of 22 nm and ζ potential 50 ± 2 mV (Figure S16).

#### AFM Analysis

**Figure 5:**
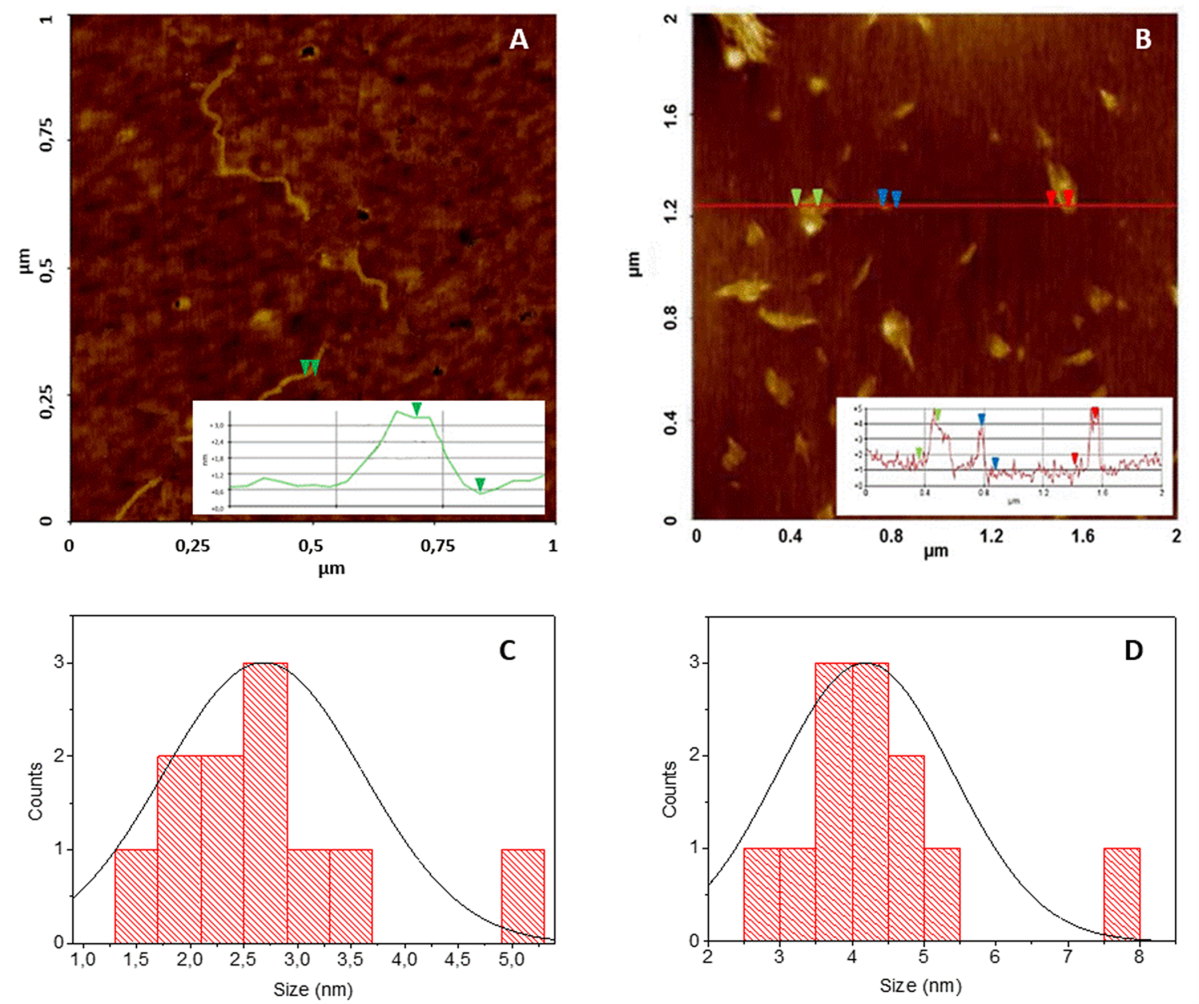
Representative images of AFM analysis for (A) Aβ_42_ alone (5 μM) and (B) copresence of **5** (5 μM) after 120 h. Statistical data for (C) Aβ_42_ (5 μM) and (D) copresence of **5** (5 μM) after 120 h.

Atomic force microscopy (AFM) studies revealed the morphologies of Aβ_42_ aggregates. Figure 5a shows that, after 120 h of incubation, Aβ_42_ (5 μM) alone formed a fibrillary network with an average fibril height of 2.65 ± 0.44 nm. The copresence of **5**, at the same concentration (5 μM), during the Aβ_42_ incubation time of 120 h, clearly inhibited the development of amyloid fibrils, and only small amorphous aggregates are detected with sizes of 4.30 ± 1.33 nm (Figure 5b). Statistical data for Aβ_42_ alone and for copresence of **5** are reported in Figure 5c and d, respectively. The AFM images further confirmed the ability of **5** to inhibit Aβ_42_ fibril formation in keeping with the spectroscopic results.

#### MS Study of Aβ_42_/**5** and Aβ_42_/GPGKLVFF Interactions

**Figure 6:**
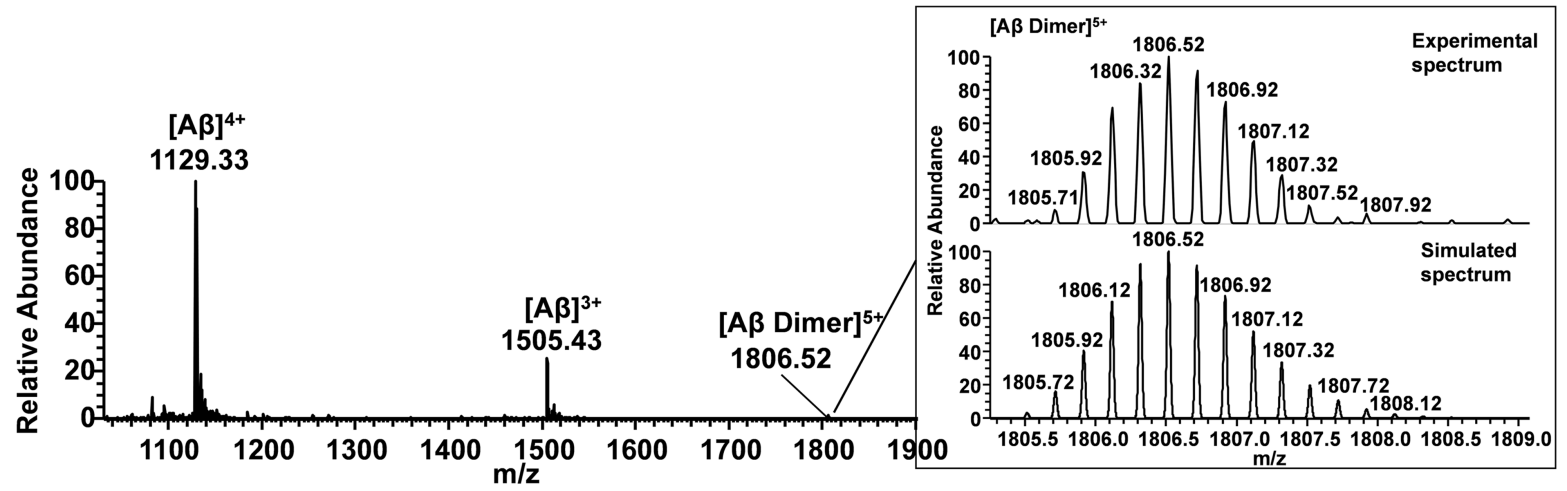
ESI-MS spectrum of Aβ_42_. The inset in the Figure shows the comparison between the experimental and theoretical isotopic distribution of peak corresponding to the Aβ_42_ dimer in the +5 charge state.

ESI-MS measurements were carried out to verify the formation of molecular adducts when GPGKLVFF and the relative conjugate **5** were added to the Aβ_42_ sample solution. The Aβ_42_ sample and the 1:5 mixtures of Aβ_42_/**5** and Aβ_42_/GPGKLVFF, were monomerized using the protocol reported in the Experimental Section and lyophilized overnight. The lyophilized sample was dissolved in hexafluoroisopropanol (HFIP) and diluted in H_2_O, to obtain a Aβ_42_ final concentration of 5.5 × 10^–6^ M (1% HFIP). The ESI-MS spectrum of Aβ_42_ reported in Figure 6 shows *m*/*z* signals at 1129.33 and 1505.43 corresponding to [Aβ_42_]^4+^ and [Aβ_42_]^3+^, respectively. A closer inspection of the ESI-MS spectrum revealed a signal at *m*/*z* = 1806.52 corresponding to the mass of the dimeric peptide in the +5 charge state (Figure 6, inset). This signal disappeared in the mass spectra acquired in the Aβ_42_/**5** sample (Figure S17), whereas *m*/*z* signals 1507.79 and 2010.06 corresponding to the +4 and +3 charge state of Aβ_42_/**5** adduct were observed. Similar effects were seen in the mass spectrum of the Aβ_42_/GPGKLVFF sample (Figure S18). Here, *m*/*z* signals 1345.20 and 1793.27 can be assigned to the [Aβ_42_/GPGKLVFF]^4+^ and [Aβ_42_/GPGKLVFF]^3+^ adducts, respectively, with concomitant loss of the dimeric form of Aβ_42_.

**Figure 7:**
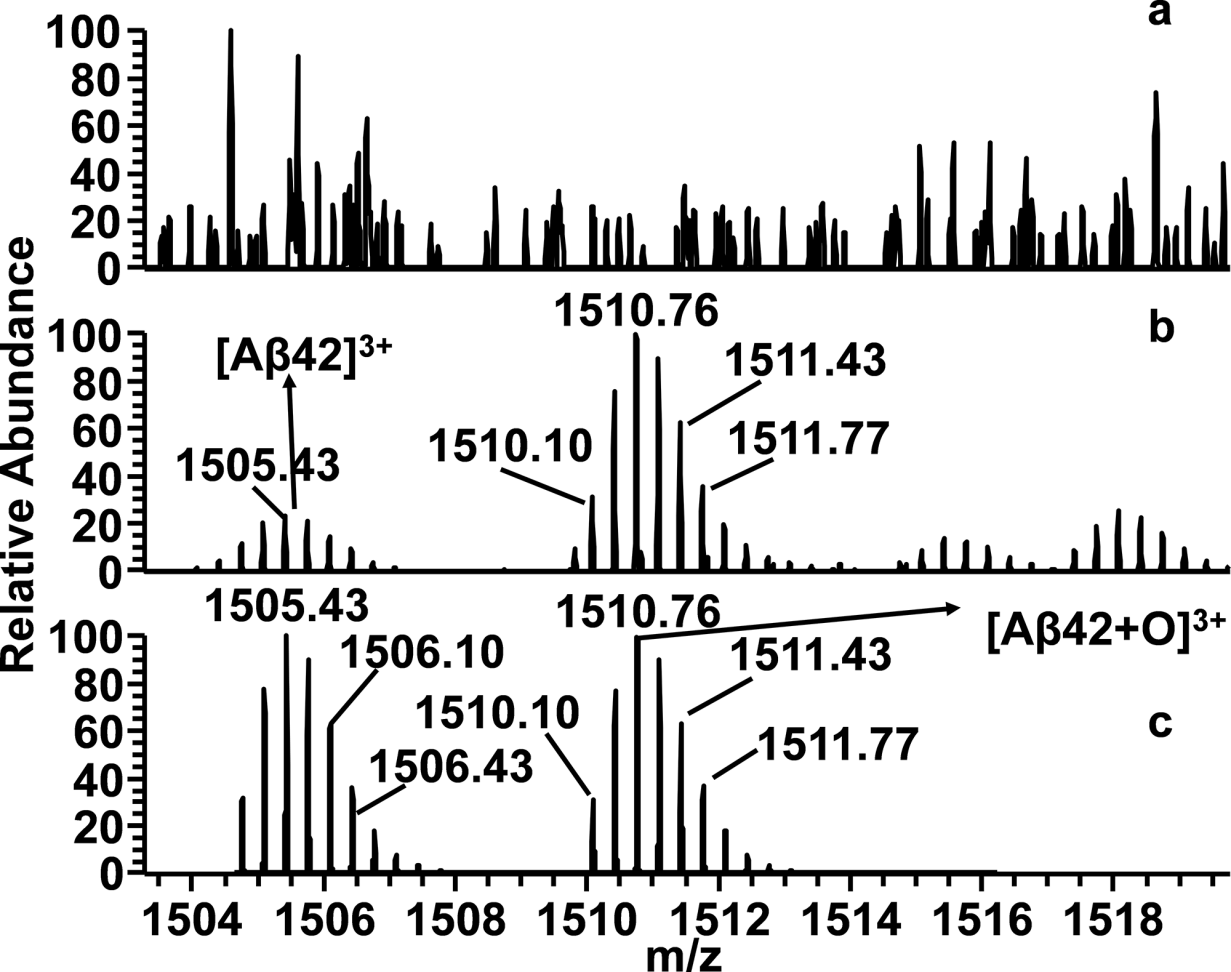
ESI mass spectra of (a) Aβ_42_ sample digested with trypsin and (b) Aβ_42_/**5** sample digested with trypsin. (c) Simulated spectrum of Aβ_42_ and oxidized Aβ_42_ form (Aβ_42_+O) for comparative analysis.

**Figure 8:**
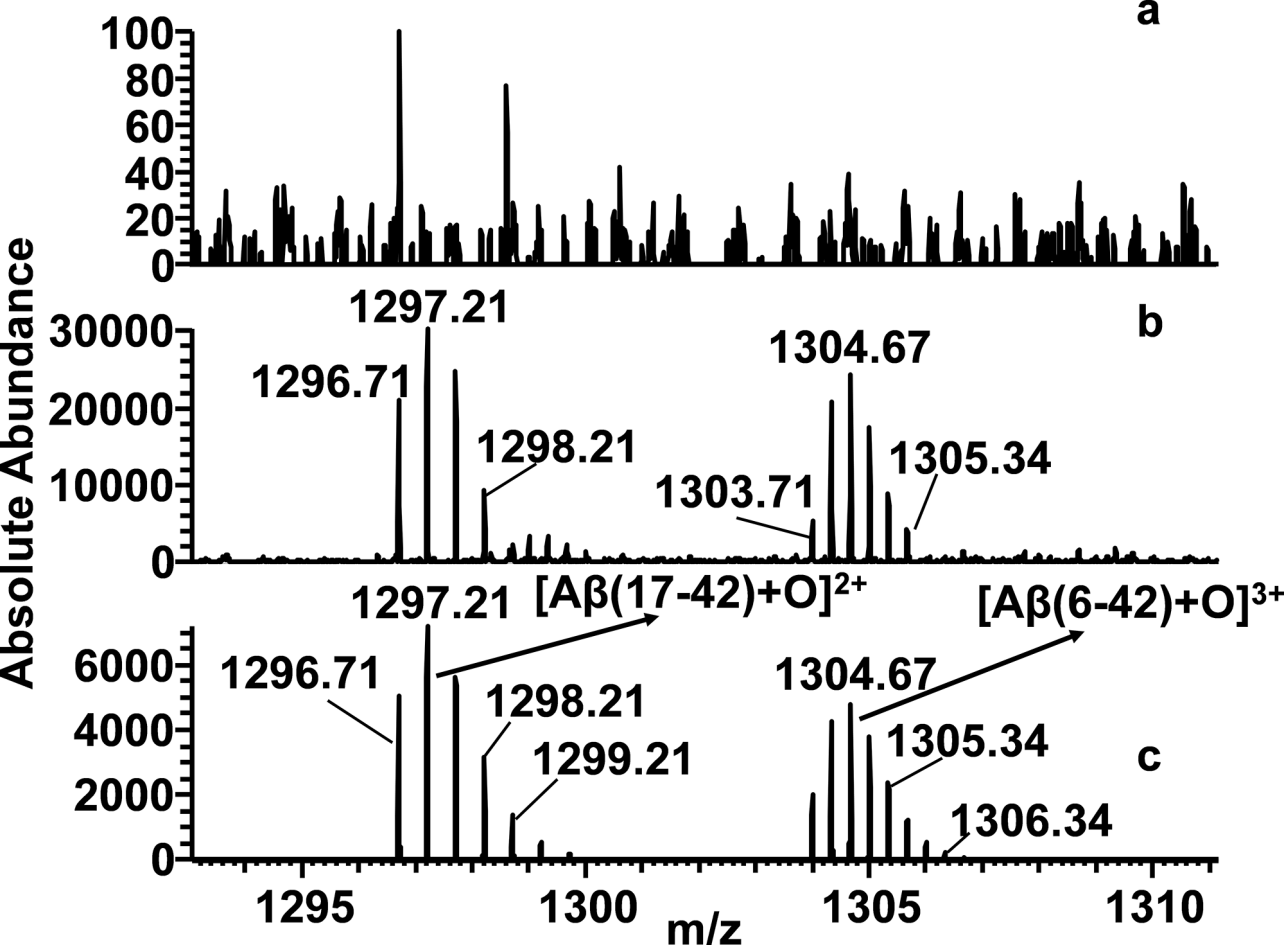
ESI mass spectra recorded at 1293–1311 *m*/*z* range of (a) Aβ_42_ sample digested with trypsin and (b) Aβ_42_/**5** sample digested with trypsin. (c) Simulated spectrum of Aβ (17–42) and Aβ (6–42) both in the oxidized form for comparative analysis.

Despite the mass spectrometric results were deduced from the gas-phase system, they nicely support what was observed in solution in terms of direct interactions between Aβ_42_ and GPGKLVFF or **5**. Nevertheless, this study reveals that the dimeric form of Aβ_42_ is no longer detectable in the MS spectrum when either GPGKLVFF or **5** is present in the sample solution. Such evidence suggests, once more, that these interactions occur in the solution phase and may play a role in the early events of the Aβ_42_ aggregation process. As reported in our previous works,^16,27^ the identification of proteolysis resistant peptides fragments by mass spectrometry may reveal the binding region of Aβ_42_ to specific molecules. Indeed, cleavage of the peptide bonds by a protease is rapid in easily accessible unstructured regions of a polypeptide, whereas the steric hindrance of other molecules at the cleavage sites should affect the rate of hydrolysis. We analyzed the Aβ_42_ peptide fragments generated after 2 h of trypsin digestion. This enzyme selectively catalyzes the hydrolysis of peptide bonds at the C-terminal side of lysyl and arginyl residues. As a matter of fact, the peptide fragments Aβ(1–5), Aβ(6–16), and Aβ(17–28) were detected in the ESI-MS spectrum (see Figure S19), indicating the cleavages expected occur at positions 5, 16, and 28 of Aβ_42_. The peptide fragment Aβ(1–16) was also observed. It could be related to the conformational features of Aβ_42_ that affect the accessibility to the Arg-5-His-6 cleavage site (Scheme S1). The absence of signals corresponding to Aβ_42_ suggested that protein digestion by trypsin was totally accomplished after 2 h (Figure 7a). Interestingly, the peptide fragments Aβ(17–42), Aβ(6–42), and full length Aβ_42_, imputable to incomplete Aβ_42_ processing, were observed in the mass spectra (Figures 7b and 8b, Scheme S2) recorded after 2 h of trypsin digestion in the copresence of **5**. Similar results were observed in mass spectra of Aβ_42_/GPGKLVFF mixture (data not shown). It is important to note that both GPGKLVFF and **5** were also subjected to trypsin degradation indicating that these peptides do not inhibit enzyme’s activity. Therefore, it can be envisioned that the interaction of GPGKLVFF or **5** with Aβ_42_ affects the processing of the amyloid protein by trypsin. Oxidized forms of Aβ and related fragments were seen in the MS spectra. Oxidation of peptides and proteins during electrospray ionization may occur because the generation of ions in ESI source is related to an electrochemical process.^53^

#### Cytotoxicity Studies

**Figure 9:**
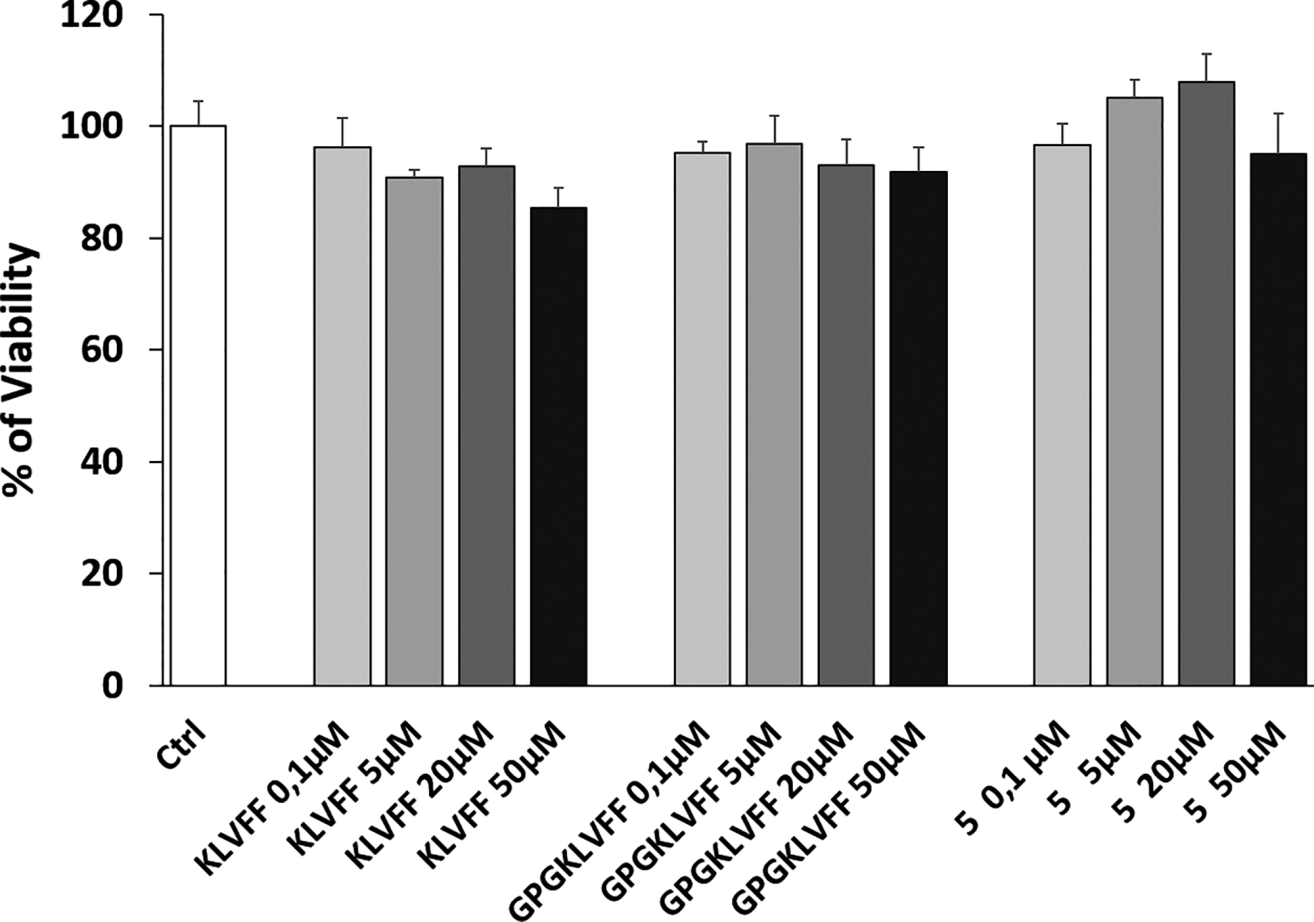
MTT assay was performed on differentiated SH-SY5Y cells after 24 h treatments. Cells were exposed to increasing concentrations of **5** (0.1, 5, 20, 50 μM). Cells were also treated with the same concentrations of appropriate controls (KLVFF or GPGKLVFF). Bars represent means of three independent experiments with *n* = 5.

Based on the previous promising results, we tested the peptide conjugate **5** for its biological activity in order to (i) exclude any potential toxicity to neuronal cells and more interestingly (ii) assess its ability to prevent oligomers toxicity. For this purpose, we used the neuroblastoma cell line, SH-SY5Y, fully differentiated according to a well-established protocol (see the Experimental Section). After prolonged treatments with all-*trans*-retinoic acid (RA) and the consequent acquisition of a neuronal-like phenotype, we exposed cells to increasing concentrations of compound **5** (0.1, 5, 20, 50 μM) for 24 h (Figure 9). The peptides KLVFF and GPGKLVFF were also added as controls. As expected, the peptide KLVFF as well as the longer peptide containing the tripeptide GPG sequence (GPGKLVFF) revealed similar activity to that of control. Interestingly, no toxicity was observed for compound **5** at any of the concentrations used, with a positive, even if not statistically significant, trend between dose (0.1–20 μM) and cellular response, after 24 h exposure. Hence, we tested the ability of compound **5** to protect neurons from the Aβ_42_ oligomer cytotoxic insult. Aβ oligomers (oAβs) were obtained from freshly prepared solution of Aβ_42_, incubated for 48 h at 4 °C in the presence or in the absence of the calixarene–peptide conjugate **5**.

**Figure 10:**
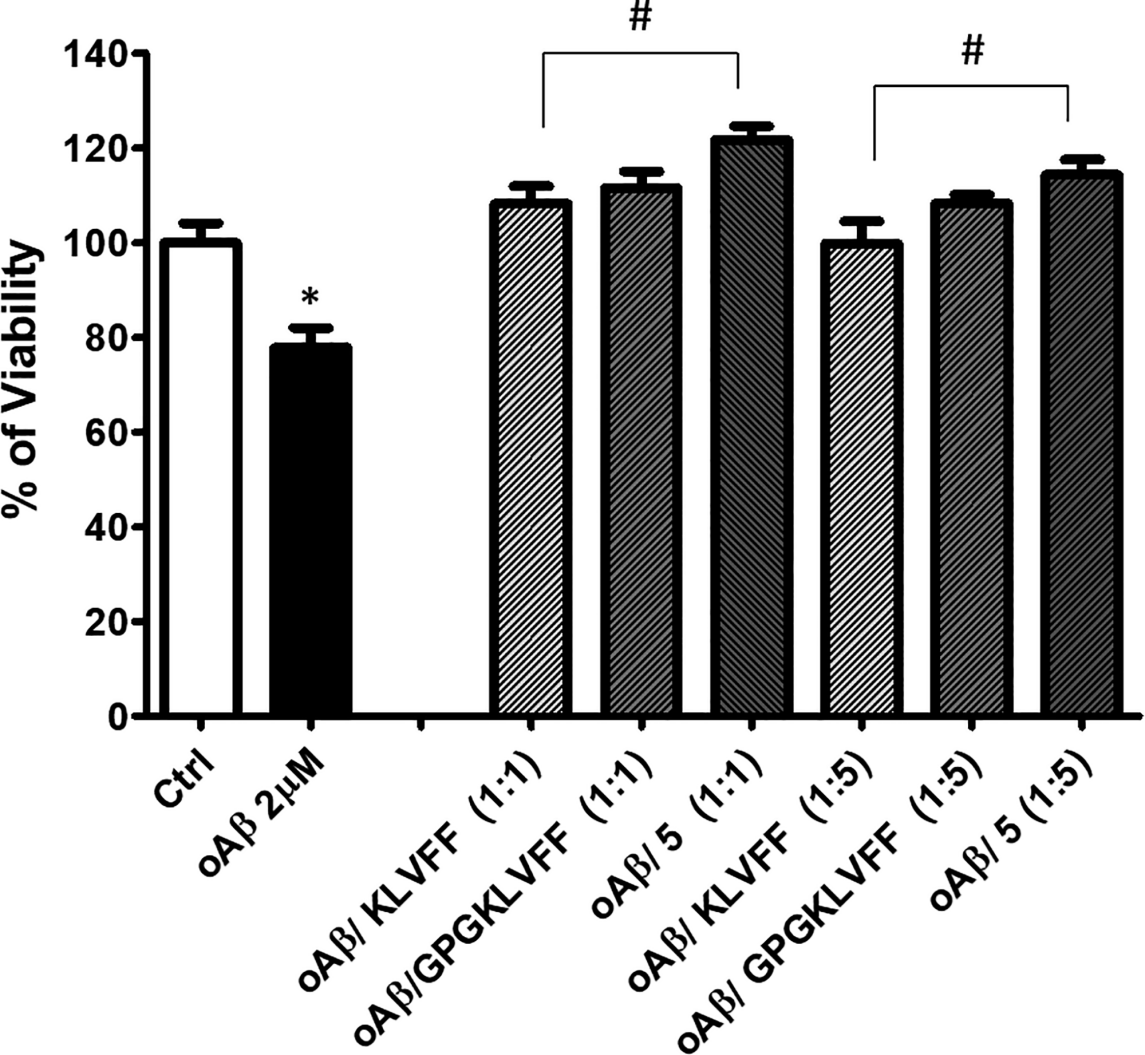
MTT assay was performed on differentiated SH-SY5Y cells after 48 h treatments. Cells were exposed to 2 μM Aβ oligomers incubated with or without compound **5** at the molar ratios of 1:1 and 1:5. Coincubations with KLVFF alone and GPGKLVFF were also performed as experimental controls. Bars represent means ± SEM of three independent experiments with *n* = 3. *P < 0.05 vs control by one-way ANOVA + Tukey test. ^#^*P* < 0.05 vs oAβ by one-way ANOVA + Tukey test.

Aβ oligomers obtained following the incubation were added at the final concentration of 2 μM, alone or in combination with all of the compounds at the molar ratios of 1:1 and 1:5. Cells, were then exposed for 48 h to each coincubated mixture and cell viability was quantified by MTT assay. As shown in Figure 10, Aβ oligomers induce a slight but statistically significant reduction of cell viability that is clearly prevented in the presence of compound **5**. We have already reported on the antioligomerization activity of a trehalose conjugated LPFFD peptide.^54^ The newly synthesized compound shows a similar interesting effect of the Aβ derived pentapeptide with a better trend, making this functionalization even more promising also in the light of calix[4]arenes ability to be potentially loaded with selected drugs. The biological effect observed on SH-SY5Y differentiated cells has been also confirmed by SDS-polyacrylamide gel experiments. The same solutions used to treat the cells were in fact loaded onto 4–12% gel polyacrylamide to assess the amount and size of Aβ_42_ aggregates formed in the presence of the compound **5** or its related controls. Two different concentrations of Aβ_42_ have been used and each peptide added with a molar ratio of 1:5. Results show that, as expected, Aβ alone during 48 h incubations at 4 °C was able to give rise to the typical migration pattern in which low molecular weight size (LMW: monomers, dimers, and tetramers) and high molecular weight (HMW: >50 kDa oligomers) size are observed (Figure S20). Coincubation with compound **5** strongly decreases the HMW band signal, which confirmed that our compound interferes with Aβ aggregation.

## Conclusion

In the present work, we have described the design, synthesis, and biophysical properties of a new calixarene–peptide construct that combines the Aβ recognition ability of the GPGKLVFF sequence with the host and recognition properties of the calixarene macrocycle. This novel compound is able to prevent cross-β-sheet elongation of Aβ_42_ through a synergistic action of both calixarene and peptide moieties that can be considered as β-sheet breaker elements. This is an interesting example of a small construct being able to preserve the nontoxic monomeric species of Aβ_42_ from being recruited into oligomeric/fibrillar aggregated forms, as demonstrated by circular dichroism and ThT fluorescence. The lack of toxicity, combined with the significant protective action on neuronal cells, further points toward a considerable therapeutic potential of this novel construct. Moreover, given the already established pharmaceutical functions of calixarenes in drug delivery, our findings suggest that the conjugate has great potential as a vehicle for the targeted delivery of additional therapeutic agents in AD. In fact, due to the presence of calix[4]arene and peptide moieties, multiple potential actions can be expected, and the possibility that the calixarene cavity offers to host and transport a variety of guests, such as drugs or imaging agents, opens up interesting perspectives for further investigations in diagnosis and therapy for amyloid related diseases. The calix[*n*]arene family offers a variety of macrocyclic scaffolds differing in size and structural conformation that, together with the versatility and ease of functionalization, may provide an additional tool for the development of other peptide–calixarene conjugates for Aβ aggregation inhibitors.

## Experimental Section

### Materials and Instrumentation

All the reagents were of analytical grade. Calix[4]arene derivative **1**(45) and GPGKLVFF peptide were prepared according to standard chemical procedures reported below. Aβ_42_ was obtained from Bachem (Switzerland). MALDI-TOF spectra were recorded on the SCIEX TOF/TOF 5800 instrument. The MALDI-MS spectra were carried out using α-CHCA as a matrix with a thin layer deposition method. Lyophilized samples (0.1 mg) were dissolved in 100 μL of 1:1:0.01 acetonitrile/water/TFA. Sinapinic acid (SIN) and α-CHCA were prepared by dissolving 4–8 mg/vial of matrices in 1 mL of 50% acetonitrile in 0.3% TFA and 1 mL of 30% acetonitrile in 0.3% TFA, respectively. Standard kits were used to calibrate the mass scale of the MALDI mass spectrometer. The Peptide Mass standard kit includes des-Arg1-bradikynin, angiotensin I, Glu1-fibrinopeptide B, ACTH (Clip 1–17), ACTH (Clip 18–39), and ACTH (Clip 7–38), and it was used to cover a mass range of 800–4000 Da.

^1^H NMR (400.13 MHz), ^13^C NMR (100.61 MHz), and 2D NMR spectra were acquired on a Bruker Avance 400 spectrometer. Chemical shifts (δ) are expressed in parts per million (ppm), referenced to the residual methanol peak; coupling constant (*J*) values are given in Hz.

#### Synthesis of GPGK(Dde)LVFF

The peptide was synthesized using the microwave assisted solid phase peptide synthesis strategy on a Liberty Peptide Synthesizer (CEM). The peptide chain assembly was carried out on a NovaSyn TGR resin (substitution 0.22 mmol/g) using the Fmoc chemistry method. All Fmoc-amino acids were introduced according to the TBTU/HOBT/DIEA or COMU activation methods. All syntheses were carried out under a 4-fold excess of amino acid. Removal of Fmoc protection during synthesis was achieved by means of 20% piperidine solution in DMF. The following instrumental conditions were used for each coupling cycle: microwave power 25 W, reaction temperature 75 °C, coupling time 300 s. The instrumental conditions used for the deprotection cycles were: microwave power 25 W, reaction temperature 75 °C, deprotection time 180 s. The peptides were cleaved off from the resin using a mixture of TFA/H_2_O/TIS (95:2.5:2.5 v/v/v). Crude peptides were recovered by precipitation with freshly distilled diethyl ether. The purification of crude GPGK(Dde)LVFF was carried out by preparative reversed-phase HPLC using a SHIMADZU LC-20A chromatography system equipped with a SPD-M20A photodiode diode array detector with detection at 222 and 254 nm. A Kinetex C18 250 × 21.10 mm (100 Å pore size, AXIA Packed) column was used. The peptides were eluted at a flow rate of 10 mL/min according to the following protocol: from 0 to 5 min isocratic conditions in 95% solvent A (H_2_O containing 0.1% TFA) followed by a 20 min linear gradient from 5 to 70% B (CH_3_CN containing 0.1% TFA) and then 5 min isocratic conditions in 70% B. Fractions containing the desired product were collected and lyophilized. Sample identity was confirmed by ESI-ORPBITRAP MASS. Calculated mass: 1026.59; observed: [M + H]^+^ = 1027.59; [M + 2H]^2+^ = 514.30.

#### Synthesis of 5,11,17,23-Tetra-Boc-amino-25,26,27-tripropoxy-28-(2-ethoxycarbonylmethoxy)-calix[4]arene (**2**)

To compound **1** (200 mg, 0.29 mmol) dissolved in passed through basic alumina CH_2_Cl_2_ (8 mL), di-*tert*-butyl dicarbonate (0.33 mL, 1.44 mmol) was added. The mixture was stirred at rt overnight, then a 5% aq NaHCO_3_ solution (50 mL) and CH_2_Cl_2_ (50 mL) were added. The organic phase was washed with 5% aq NaHCO_3_ solution (50 mL) and water (50 mL × 2). After removal of the solvent under vacuum, the solid residue was washed with hexane and recovered by filtration to give pure compound **2** (283 mg, 90%). ^1^H NMR (CDCl_3_, 297 K) δ: 0.92 (t, 3H, *J* = 7.4 Hz, propyl CH_3_), 0.98 (t, 6H, *J* = 7.4 Hz, 2 × propyl CH_3_), 1.26 (t, 3H, *J* = 7.1 Hz, OCH_2_*CH*_*3*_), 1.46, 1.49, (s, 9H each, Boc CH_3_), 1.56 (s, 18H, 2 × Boc CH_3_), 1.85 (m, 6H, *J* = 7.4 Hz, 3 × propyl *CH*_*2*_CH_3_), 3.08 and 4.37 (AX system, 4H, *J* = 13.4 Hz, 2 × ArCH_2_Ar), 3.14 and 4.57 (AX system, 4H, *J* = 13.4 Hz, 2 × ArCH_2_Ar), 3.71 (t, 4H, *J* = 7.4 Hz, 2 × propyl OCH_2_), 3.80 (t, 2H, *J* = 7.4 Hz, propyl OCH_2_), 4.17 (q, 2H, *J* = 7.1 Hz, O*CH*_*2*_CH_3_), 4.64 (s, 2H, OCH_2_CO), 6.03 (s, 2H, 2 × ArH), 6.26 (d, 2H, *J* = 3.4 Hz, 2 × ArH), 6.43 (d, 2H, *J* = 3.4 Hz, 2 × ArH), 6.81 (s, 2H, 2 × ArH).

#### Synthesis of 5,11,17,23-Tetra-Boc-amino–25,26,27-tripropoxy-28-(carbomethoxy)-calix[4]arene (**3**)

Compound **2** (100 mg, 0.075 mmol) was suspended in THF (10 mL) in the presence of water (2 mL) and KOH (50 mg, 0.89 mmol). The resulting mixture was refluxed and stirred for 3 h. The pH was adjusted to 4 with aq 1 N HCl. The product was extracted with CH_2_Cl_2_, and the organic layer was dried over Na_2_SO_4_ and evaporated to give pure compound **3** (95.37 mg, 98%). ^1^H NMR (MeOD, 297 K) δ: 0.91 (t, 3H, *J* = 7.4 Hz, propyl CH_3_), 1.02 (t, 6H, *J* = 7.4 Hz, 2 × propyl CH_3_), 1.42, 1.51, (s, 18H each, Boc CH_3_), 1.88 (m, 6H, *J* = 7.4 Hz, 3 × propyl *CH*_*2*_CH_3_), 3.12 and 4.40 (AX system, 4H, *J* = 12.7 Hz, 2 × ArCH_2_Ar), 3.18 and 4.45 (AX system, 4H, *J* = 12.7 Hz, 2 × ArCH_2_Ar), 3.73 (t, 4H, *J* = 7.4 Hz, 2 × propyl OCH_2_), 3.95 (t, 2H, *J* = 7.4 Hz, propyl OCH_2_), 4.68 (s, 2H, OCH_2_CO), 6.58 (s, 4H, 4 × ArH), 7.08 (s, 4H, 4 × ArH).

#### Synthesis of *p*-Boc-amino-calix[4]arene-GPGKLVFF-Dde Conjugate (**4**) and Deprotection to *p*-Amino-calix[4]arene-GPGKLVFF Conjugate (**5**)

To 20 mg of calixarene derivative **3** (0.018 mmol) dissolved in 2 mL of dry DMF, pyBop (16.8 mg, 0.031 mmol) and DIPEA (10.5 μL, 0.061 mmol) were added. The mixture was stirred for 2 h at room temperature, and then GPGK(Dde)LVFF (20.9 mg, 0.020 mmol) dissolved in DIPEA (5.6 μL, 0.032 mmol) was added. After stirring at room temperature for 18 h, the solvent was removed under vacuum, the solid was washed by centrifugation, three times with diethyl ether and two times with 0.01 N HCl. The residue was dried *in vacuo*. Pure compound **4** (15 mg, 0.0072 mmol, 40% yield) was obtained by preparative TLC (MeOH/CH_2_Cl_2_, 5:95 v/v). The Dde protecting group was removed from compound **4** by treatment with 2% hydrazine (1 mL), under stirring, at rt, for 30 min. After removal of hydrazine under vacuum, 20% TFA in CH_2_Cl_2_ (1 mL) was added to the solid, for removing the Boc groups. The mixture was stirred at room temperature for 2 h. The solvent was removed under vacuum, and the solid was washed three times with diethyl ether by centrifugation, to give pure compound **5** after freeze drying (11.4 mg, 95% yield). Sample identity was confirmed by MALDI-TOF mass spectrometry. Calculated mass for C_83_H_11_2N_14_O_13_: 1512.8533; observed: 1536.0288 (M + Na)^+^; 1551.9912 (M + K)^+^. ^1^H NMR (CDCl_3_/MeOD 1:3 v/v, 297 K) δ 0.70 (d, 3H, *J* = 6.7 Hz, CH_3_ Val), 0.80 (d, 3H, *J* = 6.7 Hz, CH_3_ Val), 0.87 (d, 3H, *J* = 6.5 Hz, CH_3_ Leu), 0.88 (d, 3H, *J* = 6.5 Hz, CH_3_ Leu), 0.93 (t, 6H, *J* = 7.5 Hz, 2 × CH_3_ propyl), 0.96 (t, 3H, *J* = 7.5 Hz, CH_3_ propyl), 1.27 (m, 2H, *J* = 7.5 Hz, CH_2_ Lys), 1.44–1.60 (m, 3H, CH_2_ Lys and CH Leu), 1.60–1.90 (overlapped, 10H, CH_2_ Lys, CH_2_ Leu, 3 × CH_2_ propyl), 1.90–2.00 (m, 1H, *J* = 6.7 Hz, CH Val), 2.00–2.10 (m, 2H, CH_2_ Pro), 2.13 and 2.24 (m, 1H each, CH_2_ Pro), 2.80 (m, 2H, CH_2_ Lys), 2.80–3.20 (8 dd, 2H, CH_2_ Pro), 3.20–3.43 (overlapped, 8H, 2 × ArCH_2_Ar calixarene, and 2 × CH_2_ Phe), 3.71–3.93 (m, 6H, 3 × OCH_2_ propyl), 3.96 (d, J = 6.8 Hz, 1H, Val CH), 4.10 (d, 1H, 1 × CH_2_ Gly) 4.20–4.36 (m overlapped, 2H, CH leu and CH Lys), 4.36–4.61 (overlapped, 13H, 1 × CH_2_ Gly, CH_2_ Gly, ArOCH_2_CO, 2 × ArCH_2_Ar calixarene, CH Prol, CH Leu, 2× CH Phe), 6.34 (br s, 4H, 2 × ArNH_2_ calixarene), 6.38 (s, 2H, 2 × ArH calixarene), 6.46 (br s, 2H, ArNH_2_ calixarene), 6.66 (br d, 4H, 4 × ArH calixarene), 6.70 (br s, 2H, ArNH_2_ calixarene), 6.79 (s, 2H, 2 × ArH calixarene), 7.09–7.37 (overlapped, 10H, 5 × ArH Phe). ^13^C NMR (CDCl_3_/MeOD 1:3 v/v, 297 K): 10.5, 10.8 (q), 18.9, 19.4, 21.8, 21.9 (q), 23.4 23.6, 24.0, 25.0 (d), 25.7, 27.4, 30.3, 38.2, 38.5, 40.3, 40.8, 42.4, 44.2 (t), 54.2, 54.6, 55.9, 56.5, 61.1, 62.2 (d), 74.7, 77.9 (t), 122.1, 123.2, 127.6, 127.7, 129.3, 129.4, 130.0, 130.2 (d), 136.6, 136.7, 137.1, 138.0, 138.3 (s), 169.3, 171.9, 172.1, 172.9, 173.4, 174.3, 175.1, 175.6 (s, CO).

#### Sample Preparation

Aβ_42_ was dissolved in TFA (1 mg/mL) and sonicated for 10 min. Then the TFA was removed under a gentle stream of N_2_, 1 mL of HFIP was added, and the mixture was dried under N_2_ stream to remove the remaining trace of TFA (×2). Aβ_42_ was again dissolved in 1 mL of HFIP and was frozen at −70 °C and then lyophilized overnight. The same procedure was carried out in the presence of conjugate **5**.

#### Circular Dichroism Spectroscopy

CD spectra were acquired using a J-810 spectrometer (Jasco, Japan) under a constant flow of N_2_ at room temperature. The CD spectra were recorded for Aβ_42_ (5 μM) monomer in the absence and presence of **1** and **5** (5 and 25 μM). The lyophilized samples were dissolved in 50 μL of 10 mM NaOH and then diluted with 10 mM phosphate buffer pH 7.4 containing 100 mM NaCl to 2 mL to obtain a concentration 5 μM for Aβ_42_ alone and the mixture of Aβ_42_ and conjugate **5** (1:1 and 1:5 molar ratio). A 1 cm path length quartz cuvette was used to acquire the far-UV CD spectra (190–260 nm) at a scan speed of 50 nm/min. There were 10 scans collected. The CD signal of the solution without Aβ_42_ was subtracted from the sample CD spectra. The measurements were performed in triplicate.

#### Thioflavin T Fluorescence Assay

ThT fluorescence kinetics were measured on a Flash Thermo Varioskan spectrofluorometer with excitation and emission at 450 and 480 nm, respectively. Aβ_42_ alone and in the presence of the conjugates **1** or **5** in 1:1 or 1:5 molar ratio was dissolved in 10 mM aq NaOH (30 μL). The samples were diluted (to 150 μL) with 60 μM ThT solution in 10 mM phosphate buffer at pH 7.4 to obtain a final Aβ_42_ concentration of 20 μM. The samples were incubated a 37 °C in a 96-well plate. To minimize evaporation effects the multiwell plate was sealed with a transparent heat-resistant plastic film. Readings were taken every 10 min, after weak shaking for 10 s. The fluorescence intensity was monitored for 67 h. The measurements were performed in triplicate. To minimize errors during sample preparation, we freeze dried the aliquots of monomerized Aβ_42_ and **5** directly into each well of the plate. The experimental data were fitted by using the equation:
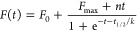
where *F*_0_ is the initial fluorescence emission and *F*_max_ is the final increment of fluorescence emission; 1/*k* is the elongation rate constant, and *t*_1/2_ is the time at which the amplitude of ThT emission is 50% of the *F*_max_ value. *t*_lag_ is defined as the intercept between the time axis and the tangent of the curve with slope *k* from the midpoint of the fitted sigmoidal curve; this parameter was calculated from the fitted parameters by using the following equation: *t*_lag_ = *t*_1/2_ – 2*k*.

The kinetic parameters are expressed as the mean (±SD) of three independent experiments.

#### Dynamic Light Scattering (DLS) and Electrophoretic Light Scattering (ELS)

DLS and ELS measurements were carried out on a ZetaSizer NanoZS90 Malvern instrument (UK), equipped with a 633 nm laser at a scattering angle of 90° and 25 °C temperature. The size of Aβ_42_, conjugate **5**, and Aβ_42_ in the presence of **5** was determined on samples prepared at the same experimental conditions as CD analyses. Each measurement was performed three times.

#### Atomic Force Microscopy

AFM analysis was performed with a PSIAXE-150 system, acquiring images of 1 × 1 and 2 × 2 μm^2^. The measurements were carried out in tapping mode using a silicon Sn doped tip (resistivity of 0.01 Ω cm and purchased by Bruker TESPA). New cantilevers were used for each measurement. Tip dimensions: thickness 4 μm, length 125 μm, width 40 μm. The stiffness was 40 N/m, and the tip was operated to an oscillation frequency of 320 kHz. An aliquot with a volume of 10 μL of sample was dispensed on a precleaned silicon flat substrate and dried. In order to perform a representative investigation of whole samples, after deposition the substrates were not rinsed with Milli-Q water (as commonly reported in literature). For each sample, various areas on the sample were investigated and statistically relevant images were chosen.

#### Mass Spectrometry Analyses

The lyophilized Aβ_42_ was dissolved in HFIP (hexafluoroisopropanol) to obtain a concentration of 2.2 × 10^–4^ M (Aβ_42_ stock solution). The GPGKLVFF peptide and compound **5** were dissolved in HFIP to obtain a stock solution of 1.1 × 10^–3^ M for each. The Aβ_42_ sample and the mixtures of Aβ_42_/GPGKLVFF and Aβ_42_/5 at 1:5 ratios, were prepared from stock solutions to a final concentration [Aβ_42_] = 5.5 × 10^–6^ M, [**5**] = [GPGKLVFF] = 27.5 × 10^–6^ M in Milli-Q water at physiological pH. For proteolysis experiments, 25 × 10^–6^ g of trypsin enzyme was dissolved in HCl 1 × 10^–3^ M [trypsin] = 2.14 × 10^–5^ M, and then an appropriate volume of the enzyme stock solution was added to the Aβ_42_, Aβ_42_/GPGKLVFF, and Aβ_42_/**5** samples to obtain a final enzyme/substrate ratio of 1:20 w/w. Solutions were incubated at 37 °C, and then the digestion reactions were stopped after 2 h by adding 1 μL 0.3% aqueous TFA. The samples were analyzed by using an ESI-MS Orbitrap Q-exactive system (Thermos Scientific). Each sample was introduced into the ESI source on 100 mm internal diameter fused silica via a 500 mL syringe. Full MS scans in the *m*/*z* range 400–4000 were acquired in the positive ion mode, spray voltage = 3.5 kV, capillary temperature = 300 °C; *m*/*z* range = 400–4000, S-lens RF level = 60 V, sheath gas = 7, resolving power: 70 000 fwhm. The spectra, recorded as raw files, were imported in Qual Browser (Thermo Scientific) software for analysis. Averaged MS spectra were imported into a freely available open-source software, mMass (http://www.mmass.org). Theoretical *m*/*z* values of Aβ_42_, GPGKLVFF, **5**, the adducts Aβ_42_/GPGKLVFF and Aβ_42_/**5**, and the peptides resulting from in silico digestion of Aβ_42_ were compared with the *m*/*z* values assigned to experimental mass spectra. Peptides that matched successfully, within a tolerance of 0.005 Da, were annotated.

#### Cell Cultures and MTT Assay

The neuroblastoma (NB) cell line, SH-SY5Y, was maintained in DMEM-F12 (Gibco, ThermoFisher) supplemented with 10% heat inactivated (HI) fetal calf serum (Gibco, ThermoFisher), 100 mg/mL penicillin and streptomycin (Gibco, ThermoFisher), and 2 mM l-glutamine at 37 °C, 5% CO_2_. Two weeks before experiments, 5 × 10^3^ cells were plated on 96-well plates in DMEM-F12 with 5% HI fetal calf serum. The percentage of serum was gradually decreased until it was 1% of the total. All-*trans*-retinoic acid (RA) (Sigma), 5 μM, was used to promote neuronal differentiation, and medium-containing RA was changed every 3 days. Treatments with compound **5**, KLVFF, and GPGKLVFF were performed on fully differentiated cells. After 24 h treatment, cultures were incubated with MTT (0.5 mg/mL) for 2 h at 37 °C and then lysed with DMSO, and the formazan production was evaluated in a plate reader through the absorbance at 570 nm.

#### Antioligomerization Assay

Aβ_42_ oligomers were prepared as previously described^54^ from synthetic Aβ_42_ following a protocol for monomerization. An amount of 1 mg of Aβ_42_ was first dissolved in 5 mM DMSO. A solution of 10 μM Aβ_42_ in ice-cold DMEM F-12 without Phenol Red was prepared and allowed to oligomerize for 48 h at 4 °C according to the Lambert protocol^55^ with some modification as previously described.^56^ In order to evaluate the ability of compound **5** and the other appropriate controls (KLVFF, GPGKLVFF), samples of Aβ_42_ (10 μM) were incubated in the presence or absence of each compound (Aβ/ligand ratios 1:1, 1:5). After 48 h incubation, Aβ/ligand compounds were applied to the differentiated SH-SY5Y cells at the final concentration of 2 μM Aβ.

#### Western Blot Analysis

A stock aliquot of 5 mM Aβ previously dissolved in DMSO was diluted in DMEM F-12 without Phenol Red, and two different samples at 20 μM and 50 μM concentrations were prepared. From each solution, two sets of samples were obtained by combining Aβ_42_ with KLVFF peptide, GPGKLVFF, or compound **5** with a molar ratio Aβ/peptide of 1:5. All the samples, including Aβ alone at the two concentrations used, were incubated at 4 °C for 48 h to form Aβ oligomers. After incubation, the amount and size of Aβ aggregates were determined by Western blot analysis. A volume of 25 μL of each unheated sample was loaded onto a precast Bis-Tris gel (Bolt 4–12%, Life Technologies) with 2-morpholin-4-yl ethanesulfonic acid (MES). Samples were transferred onto a nitrocellulose membrane (0.2 mm, Hybond ECL, Amersham Italia) by using a wet transfer unit Mini Blot Module (Life Technologies). Membranes were blocked in Odyssey blocking buffer (Li-COR Biosciences) and incubated at 4 °C overnight with mouse monoclonal anti-amyloid-β antibody against N-terminal 1–16 peptide (1:1000) (Covance). A secondary goat anti-mouse antibody labeled with IR dye 800 (1:25 000) was used at rt for 45 min. Hybridization signals were detected with the Odyssey CLx infrared imaging system (LI-COR Biosciences, Lincoln, NE).

## Supporting Information Available

Tables containing values of random coil secondary structures and ThT kinetic parameters, MS, NMR, CD, ThT spectra, DLS and ELS measurements; Western blot analysis (PDF)
