# Supplementary material for: Novel Peptide-Calix[4]arene Conjugate Inhibits Aβ Aggregation and Rescues Neurons from Aβ’s Oligomers Cytotoxicity In Vitro
Source: ACS Chem Neurosci. 2021 Apr 12;12(8):1449–62. doi: 10.1021/acschemneuro.1c00117 (PMC9535895; doi:10.1021/acschemneuro.1c00117)
Supplement: Supplementary file 1 — cn1c00117_si_001.pdf [file cn1c00117_si_001.pdf]

Supporting Information

Novel Peptide-Calix[4]arene Conjugate Inhibits Aβ Aggregation and Rescues Neurons from Aβ's Oligomers Cytotoxicity *in vitro*

Grazia Maria Letizia Consoli,* Rita Tosto, Ausilia Baglieri, Salvatore Petralia, Tiziana Campagna, Giuseppe Di Natale, Stefania Zimbone, Maria Laura Giuffrida, Giuseppe Pappalardo.*

Grazia Maria Letizia Consoli; **Ausilia Baglieri** - CNR-Institute of Biomolecular Chemistry, Via P. Gaifami 18, 95126 Catania, Italy.

Rita Tosto - International PhD School of Chemical Sciences, University of Catania, V.le A. Doria 6, 95125 Catania, Italy; CNR-Institute of Crystallography, Via P. Gaifami 18, 95126 Catania, Italy.

Salvatore Petralia - Department of Drug Sciences and Health, University of Catania, V.le A. Doria 6, 95125 Catania, Italy.

Giuseppe Pappalardo; Tiziana Campagna; Giuseppe Di Natale; Maria Laura Giuffrida; Stefania Zimbone.- CNR-Institute of Crystallography, Via P. Gaifami 18, 95126 Catania, Italy.

TABLE OF CONTENTS

Table S1. Deconvoluted values of random coil secondary structure reported as the mean value obtained with CONTIN and CDSSTR algorithms standard error (RMSD) in parenthesis.

Table S2. ThT kinetic parameters related to the aggregation of $A\beta_{42}$ alone or in the presence of the compounds **1** or **5** or GPGKLVFF, with Aβ/compound ratio 1:1 or 1:5.

Scheme S1. $A\beta_{42}$ proteolytic pattern after 2 h of trypsin digestion at an enzyme/substrate ratio of 1:20 w/w.
Scheme S2. $A\beta_{42}$ proteolytic pattern after 2 h of trypsin digestion of $A\beta_{42}$/**5** sample at an enzyme/substrate ratio of 1: 20 w/w.

Figure S1. ESI-MS spectrum of GPGK(Dde)LVFF.

Figure S2. FT-MS Spectrum of conjugate **5**.

Figure S3. HCD spectra and fragmentation pattern of conjugate **5**.

Figure S4. MALDI-TOF spectrum of conjugate **5**.

Figure S5. ^{1}H NMR spectrum of conjugate **5**.

Figure S6. 2D-COSY NMR spectrum of conjugate **5**.

Figure S7. ^{13}C NMR spectrum of conjugate **5**.

Figure S8. ^{13}C DEPT NMR spectrum of conjugate **5**.

Figure S9. 2D-HSQC-NMR spectrum of conjugate **5**.

Figure S10. 2D-HMBC-NMR spectrum of conjugate **5**.

Figure S11. CD spectra of conjugate **5** at 5 μM and 25 μM.

Figure S12. CD spectra of $A\beta_{42}$ in the presence of compound **1** (ratio 1:1 and 1:5) and compound **1** alone.

Figure S13. CD spectra of Aβ42/GPGKLVFF (ratio 1:1 and 1:5) and GPGKLVFF alone.

Figure S14. ThT fluorescence kinetic of **5** and **1** and GPGKLVFF.

Figure S15. DLS size distributions by number % for conjugate **5** at t=0 and after 120 h incubation.

Figure S16. DLS size distribution and zeta potential (ξ) of **5** at 20 μM.

Figure S17. ESI-MS spectrum of $A\beta_{42}$/**5** sample.

Figure S18. ESI-MS spectrum of $A\beta_{42}$/GPGKLVFF sample.

Figure S19. Isotopic patterns of peptide fragment produced after 2 h of $A\beta_{42}$ digestion with trypsin.

Figure S20. Representative western blot showing two different concentrations of Aβ oligomers obtained after 48 h incubation at 4°C in the presence or absence of KLVFF peptide, GPGKLVFF and compound **5**.

Table S1. Values of random coil secondary structure determined as the average of CONTIN and CDSSTR methods with deconvolution algorithms and the relative standard error (RMSD).

**Sample**	**CONTIN /CDSSTR**	**RMSD**
Aβ 0h	0.549	0.08
Aβ, 120 h	0.246	0.100
Aβ:5 1:1, 0 h	0.638	0.168
Aβ:5 1:1, 120 h	0.587	0.139
Aβ:5 1:5, 0 h	0.446	0.112
Aβ:5 1:5, 120 h	0.470	0.172
Aβ:1 1:1, 0 h	0.399	0.164
Aβ:1 1:1, 120 h	0.366	0.216
Aβ:1 1:5, 0 h	0.361	0.231
Aβ:1 1:5, 120 h	0.414	0.089
Aβ:GPGKLVFF 1:1, 0 h	0.564	0.121
Aβ:GPGKLVFF 1:1, 120 h	0.293	0.220
Aβ:GPGKLVFF 1:5, 0 h	0.508	0.344
Aβ:PGKLVFF 1:5, 120 h	0.322	0.156

Table S2. Kinetic parameters related to the aggregation of Aβ_{42} alone or in the presence of the compounds **1** or **5** or GPGKLVFF, with Aβ/compound 1:1 or 1:5 molar ratio. * Not fitted

	**Aβ (20 µM)**	**Aβ/5 (1:1)**	**Aβ/5 (1:5)**	**Aβ/1 (1:1)**	**Aβ/1 (1:5)**	**Aβ/GPGKLVFF (1:1)***	**Aβ/GPGKLVFF (1:5)**
F_{max}	10.70 ± 0.04	3.45 ± 0.02	0.53 ± 0.13	9.25 ± 0.25	4.95 ± 0.03	n.f.	7.34 ± 0.04
K	2.71 ± 0.15	3.95 ± 0.19	6.32 ± 4.73	15.69 ± 1.33	10.12 ± 0.38	n.f.	1.19 ± 0.19
$t_{1/2}$	7.13 ± 0.18	21.19 ± 0.22	46.91 ± 5.83	27.06 ± 1.08	19.92 ± 0.51	n.f.	5.54 ± 0.23
t_{lag}	1.7 ± 0.12	14.25 ± 0.16	34.27 ± 3.63	-4.32 ± 1.58	-0.30 ± 0.26	n.f.	3.16 ± 0.15

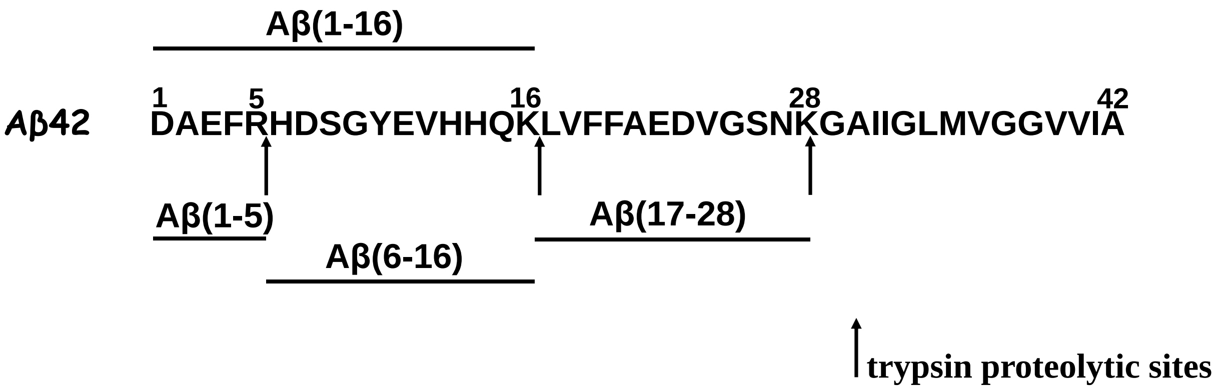

Scheme S1. Aβ_{42} proteolytic pattern after 2 h of trypsin digestion at an enzyme/substrate ratio of 1:20 w/w.

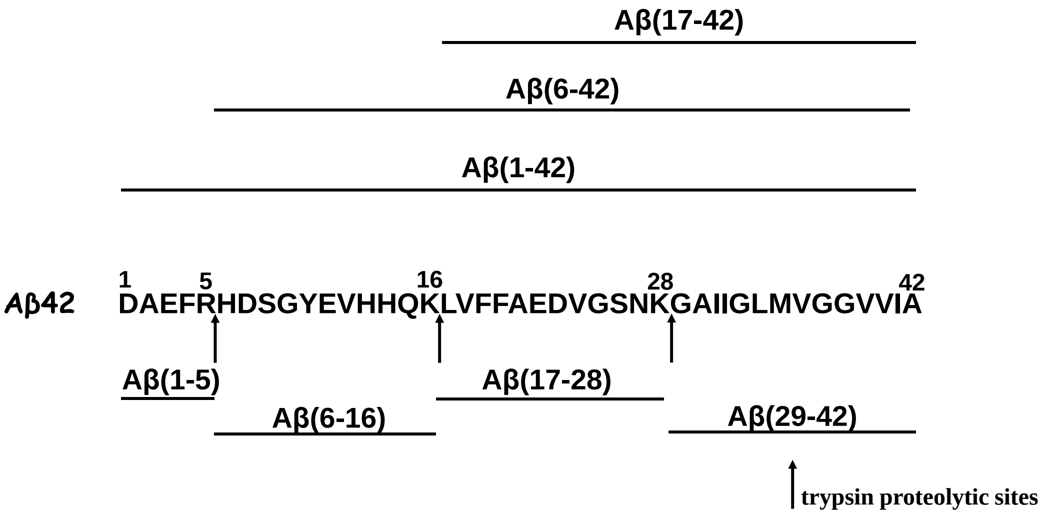

Scheme S2. Aβ_{42} proteolytic pattern after 2 h of trypsin digestion of Aβ_{42}/**5** sample at an enzyme/substrate ratio of 1: 20 w/w.

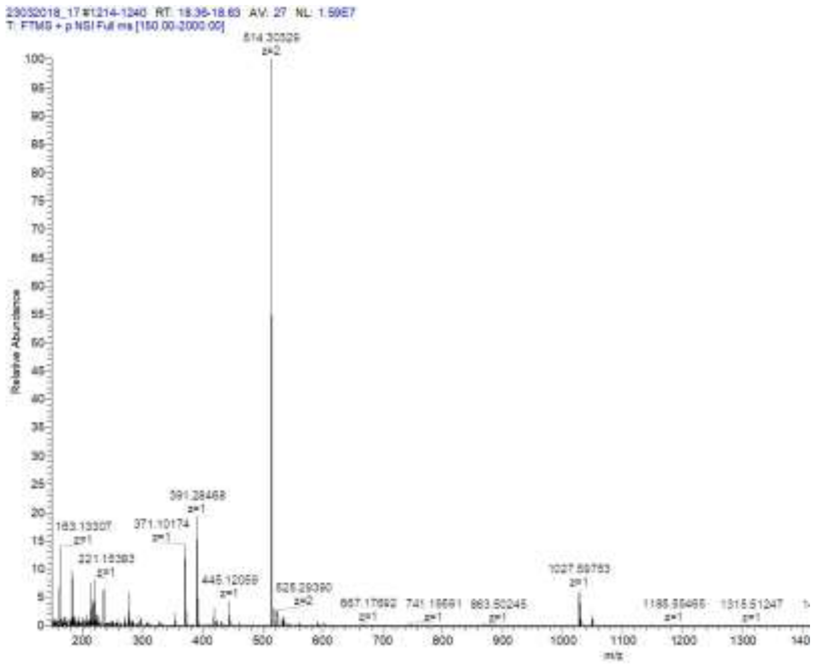

Figure S1. ESI-MS spectrum of GPGK(Dde)LVFF.

Calix[4]arene-GPGKLVFF (5) characterization

Fourier Transform (FT-MS) and tandem mass spectrometry investigations provided additional information as a completion of **5** characterization. In particular, the accuracy of the *m/z* signal assigned to the synthesised product (< 3ppm) and superimposition of the experimental and simulated isotopic distribution spectra, calculated by molecular formula, confirmed the identity of the synthesised product (**Figure S2**). Moreover, the fragmentation pattern observed in the MS/MS spectra of **5** (**Figure S3**), supported the correct amino acid sequence of the molecule. The principal fragments observed, when the *m/z* signal assigned to the **5** was selected as precursor ion, were generated by the cleavage of amide bonds in the peptide chain according to the mobile proton model theory. These fragments were labelled b_n and y_n depending on where the positive charge resides, at the N- or C-terminal side respectively. (B. Palzs, S. Suhal, *Mass Spectrom. Rev*. **2005**, *24*, 508–548) Tandem mass spectra also show internal fragments resulting from b_n and y_n fragments ions undergoing a second dissociation.

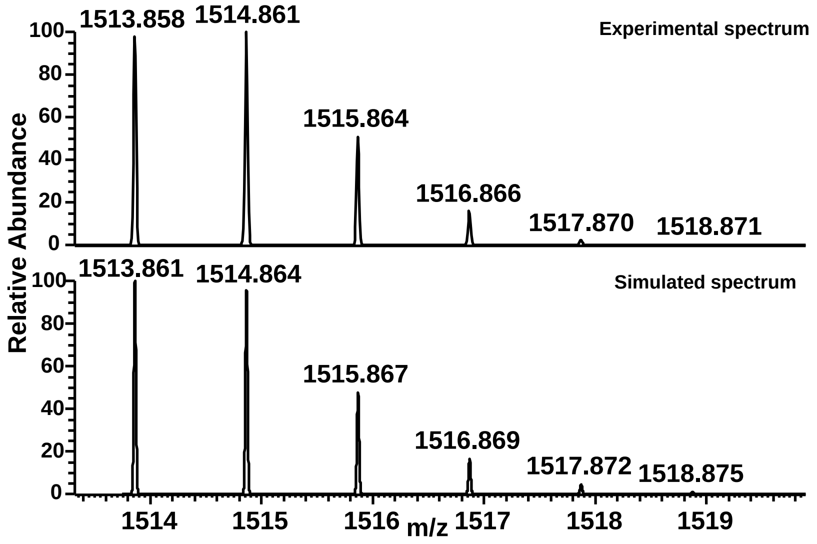

Figure S2. Measured isotopic profiles and theoretical isotopic patterns of **5**.

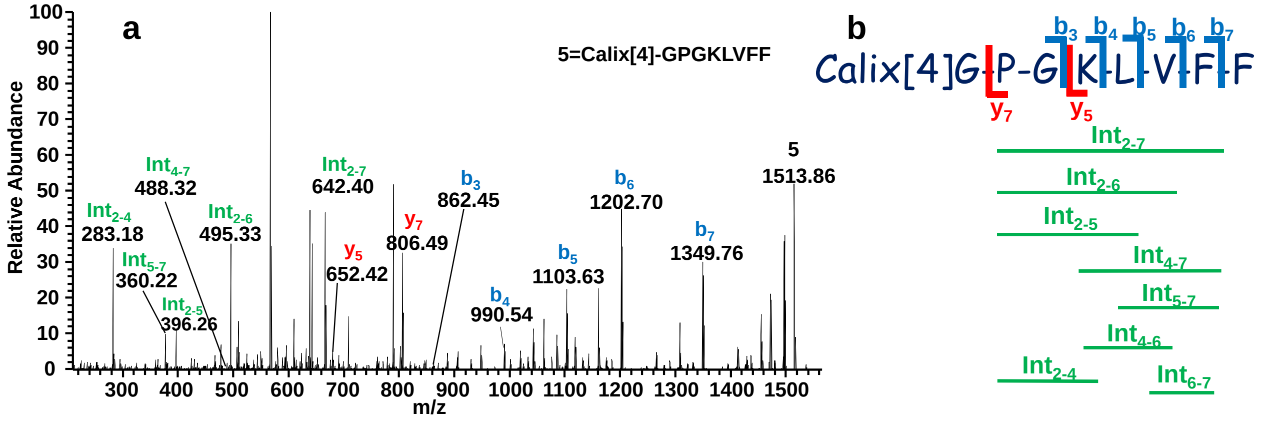

Figure S3. a) HCD spectra of single charged **5** (C_5= 27.5x10^{-6}M) (m/z $[5]^+$ = 1513.86). The b_n type fragment (blue colour), y_n type fragments (red colour) and internal type fragments (green colour) produced through the cleavage of a bond in the peptide chain were also reported; **b)** fragmentation pattern observed in the MS/MS mass spectrum reported in a).

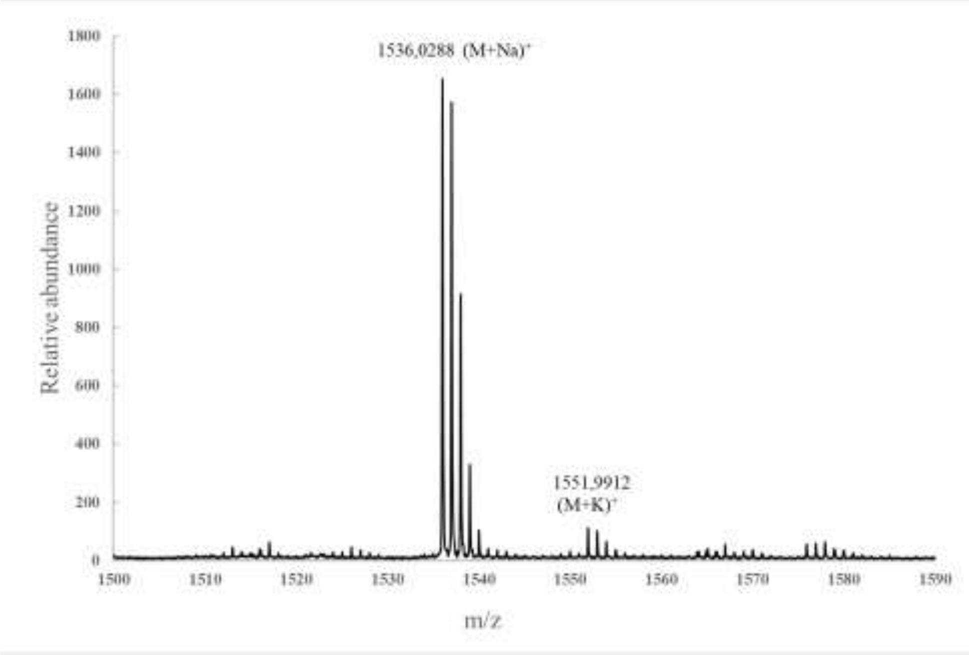

Figure S4. MALDI-TOF spectrum of conjugate **5**.

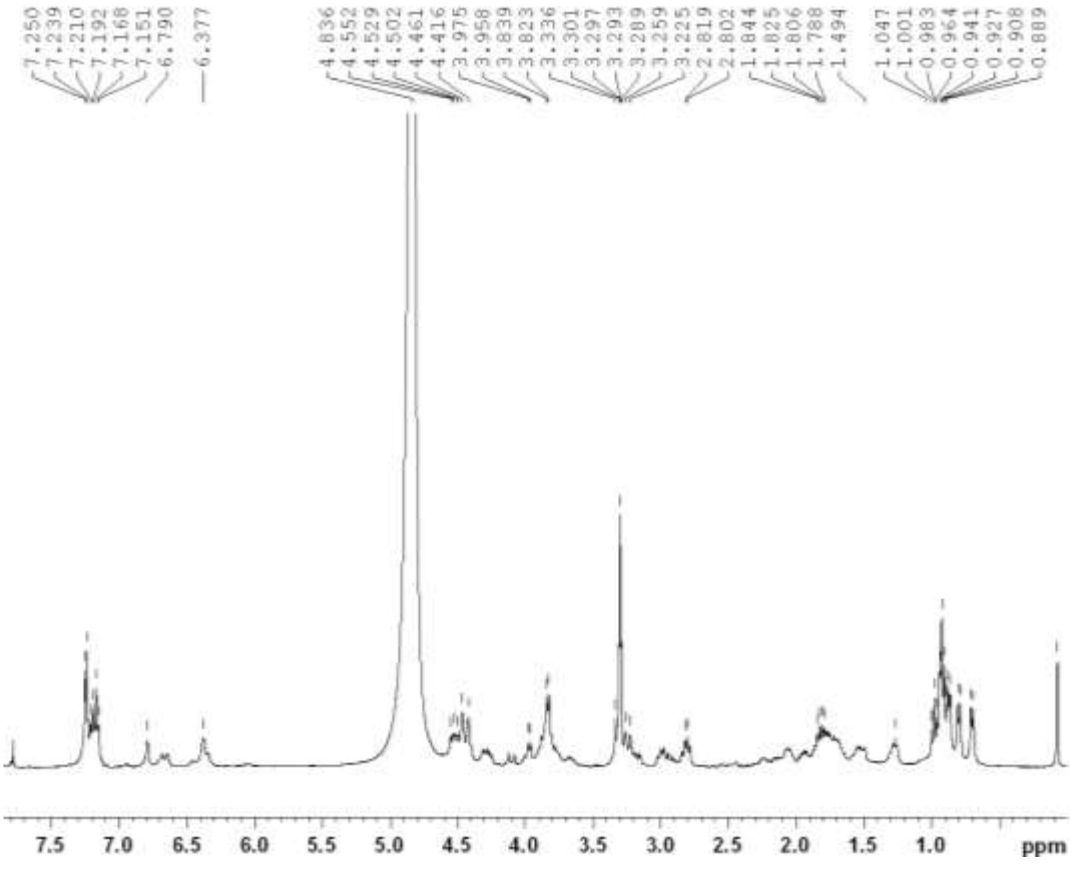

Figure S5. ^{1}H-NMR spectrum of the conjugate **5** (400.13 MHz, MeOD/$CDCl_3$ 3:1 *v/v*, 297 K).

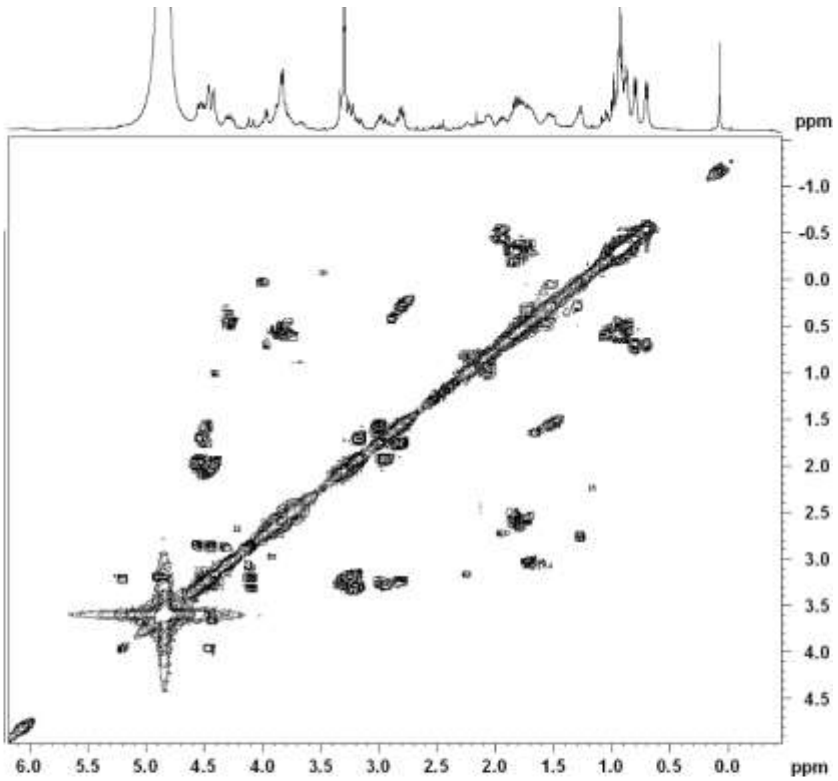

Figure S6. 2D-COSY NMR spectrum of conjugate **5** (400.13 MHz, MeOD/$CDCl_3$ 3:1 *v/v*, 297 K)

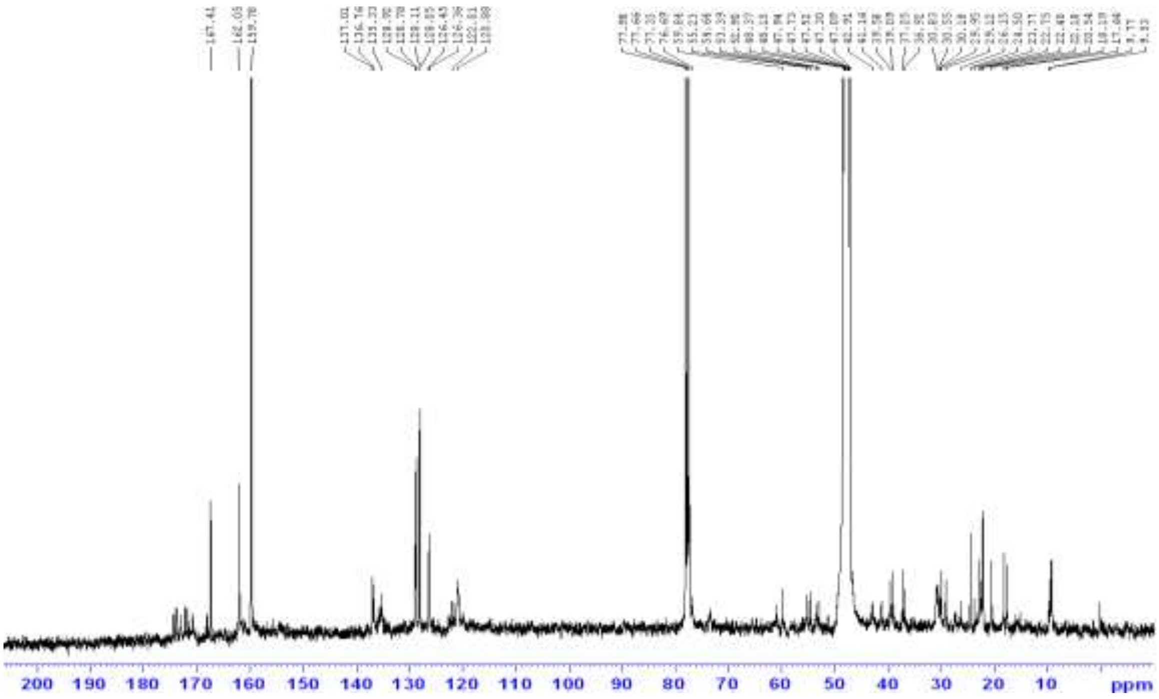

Figure S7. ^{13}C-NMR spectrum of conjugate **5** (100.61 MHz, MeOD/$CDCl_3$ 3:1 *v/v*, 297 K).

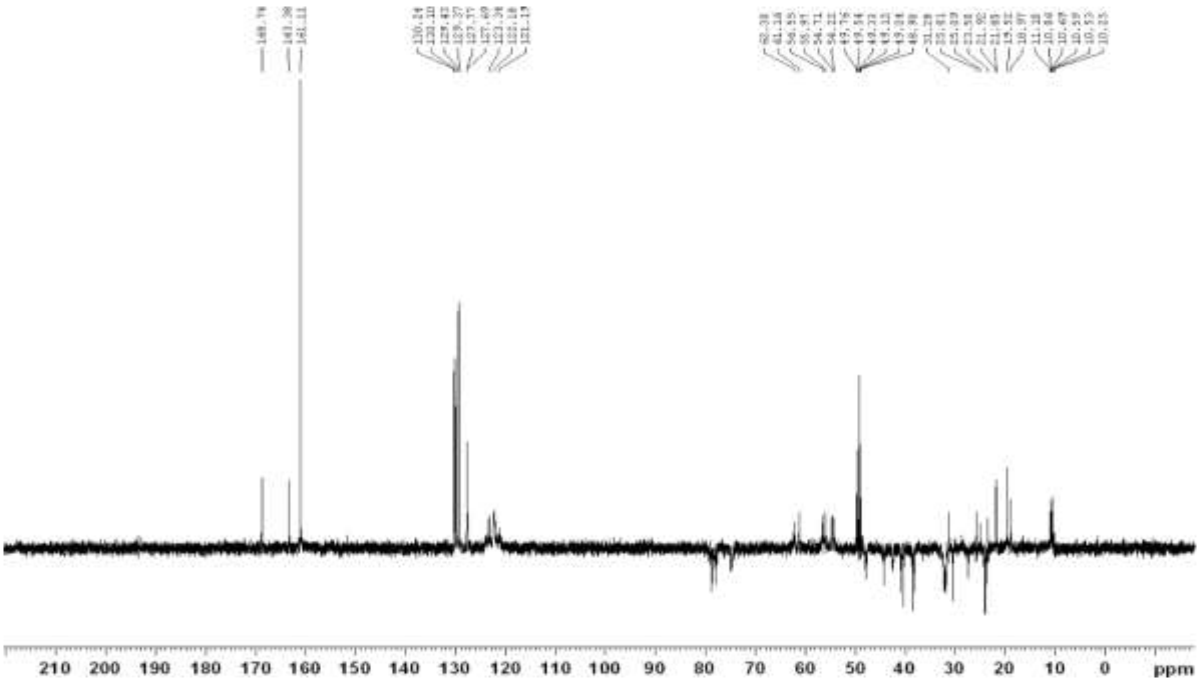

Figure S8. ^{13}C-DEPT NMR spectrum of conjugate **5** (100.61 MHz, MeOD/$CDCl_3$ 3:1 *v/v*, 297 K)

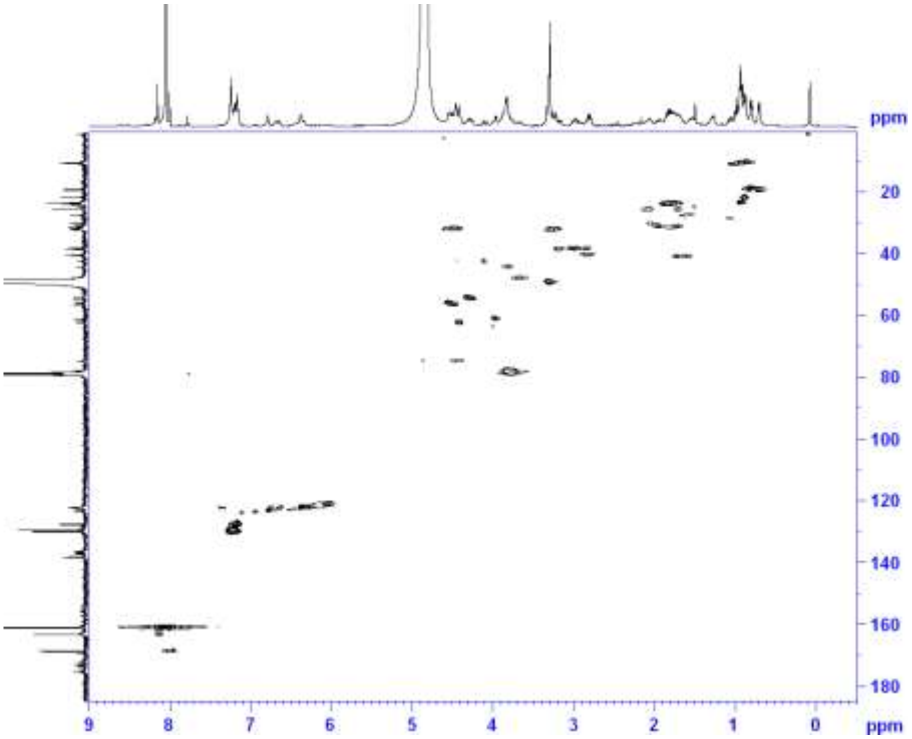

Figure S9. 2D-HSQC NMR spectrum of conjugate **5** (MeOD/$CDCl_3$ 3:1 *v/v*, 297 K)

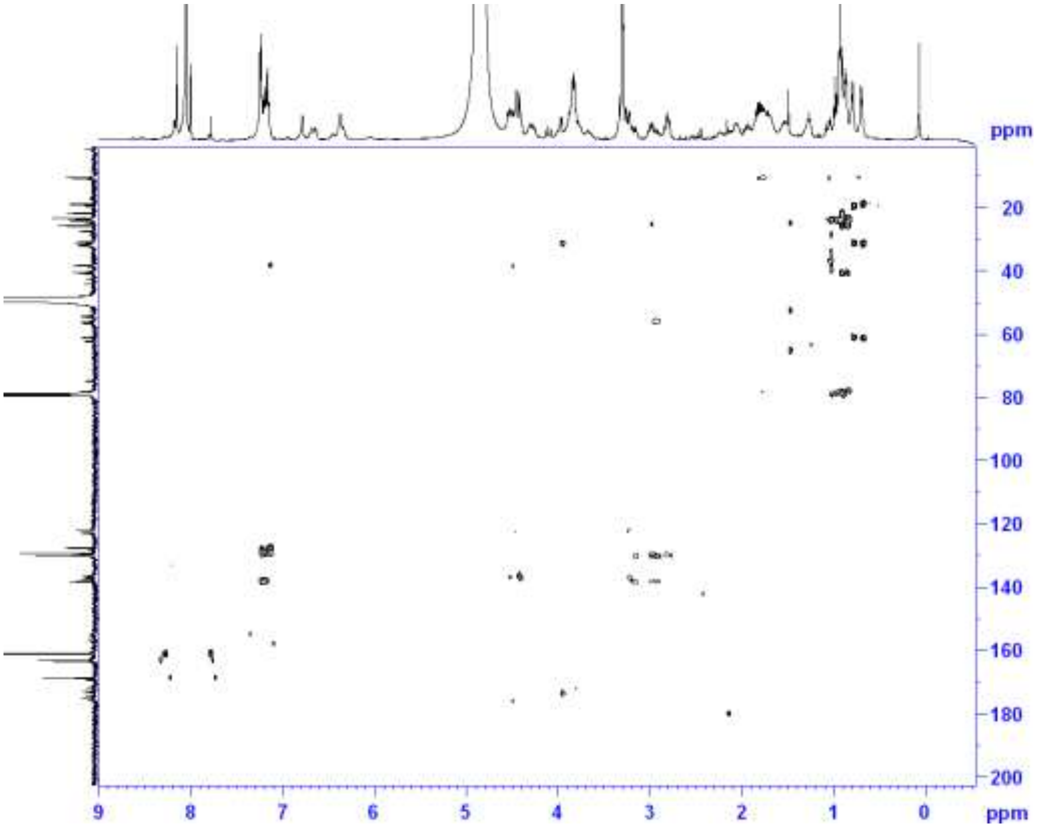

Figure S10. 2D-HMBC NMR spectrum of conjugate **5** (MeOD/$CDCl_3$ 3:1 *v/v*, 297 K)

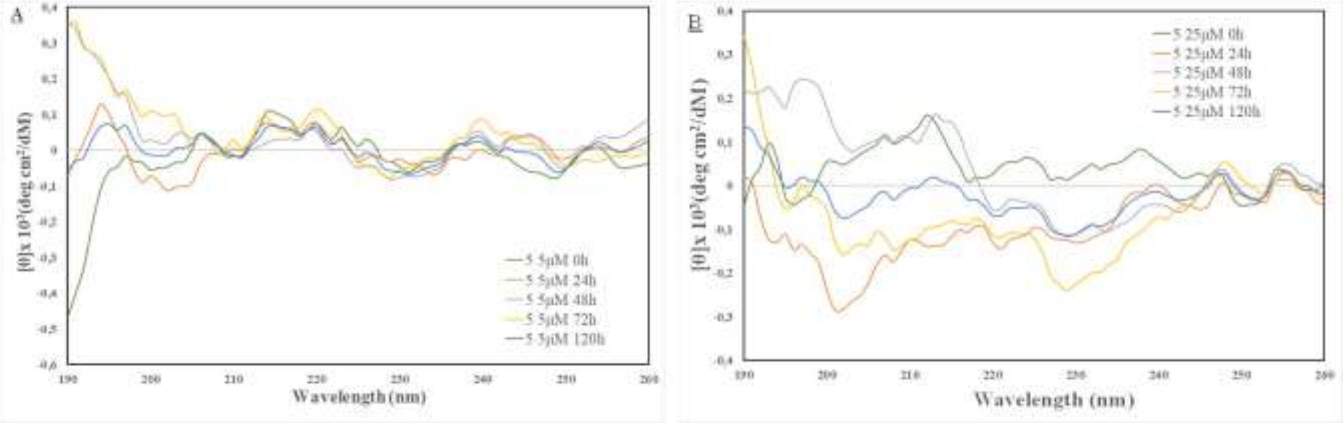

Figure S11. CD spectra of **5** (a) 5μM and (b) 25μM.

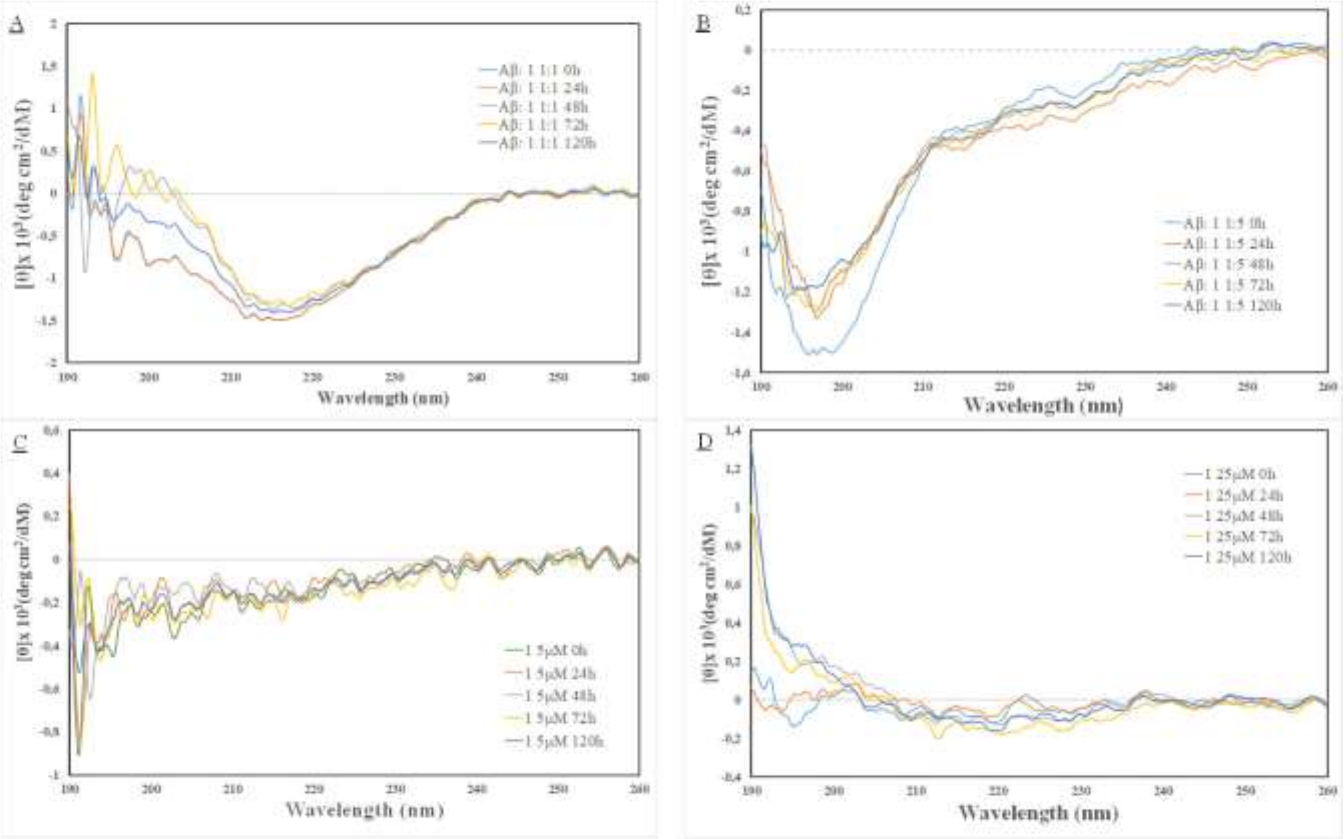

Figure S12. CD spectra of (a) Aβ_{42}/**1** (1:1 molar ratio); (b) Aβ_{42}/**1** (1:5 molar ratio); (c) **1** (5 μM); (d) **1** (25 μM).

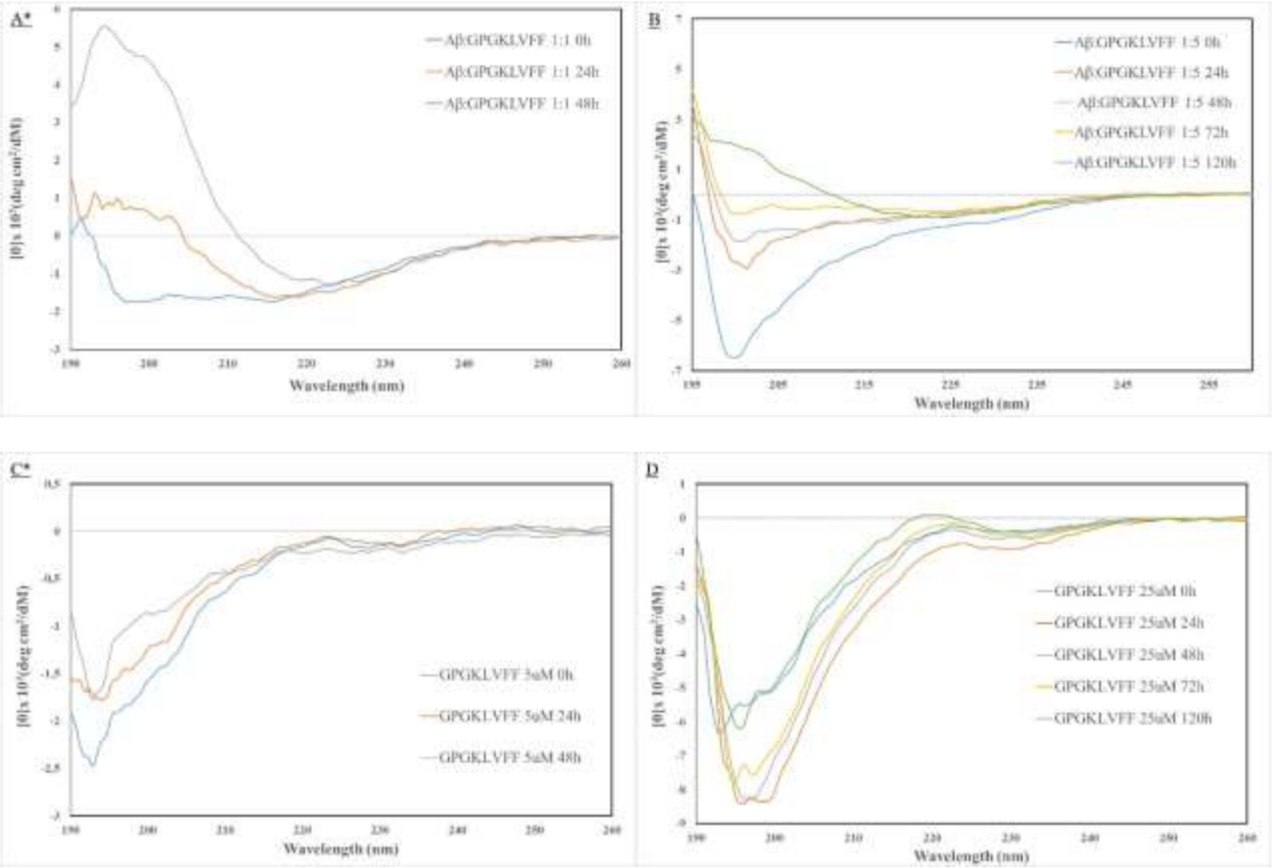

Figure S13. CD spectra of (a) $A\beta_{42}$/GPGKLVFF (1:1 molar ratio); (b) $A\beta_{42}$/GPGKLVFF (1:5 molar ratio); (c) GPGKLVFF (5 μM); (d) GPGKLVFF (25 μM). *Monitoring at 48h because of massive sample flocculation.

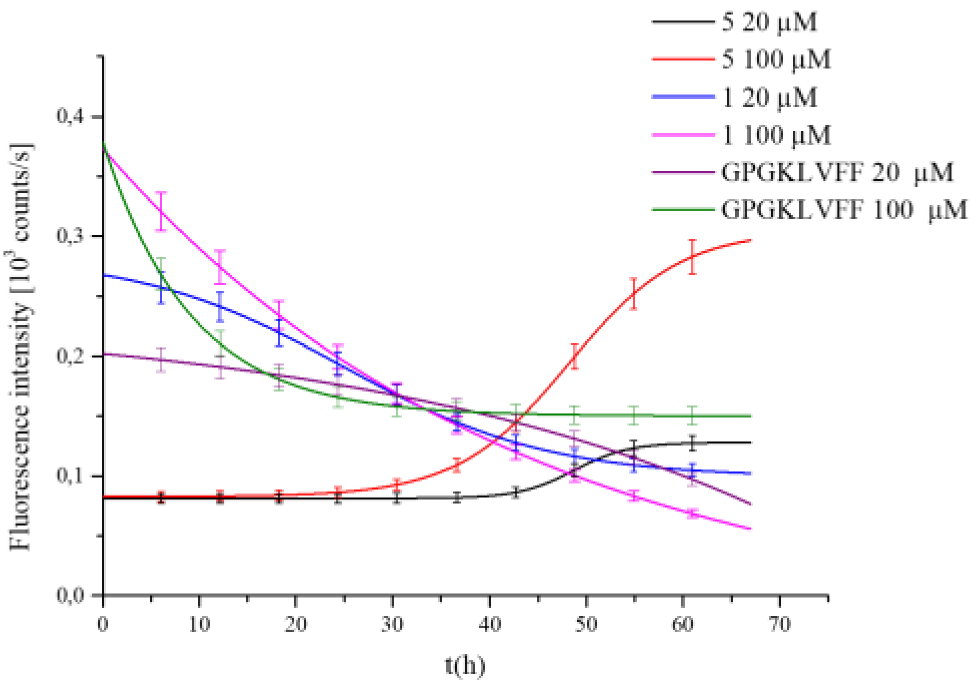

Figure S14. ThT fluorescence assays of **5** (20 µM black and 100 µM red) and of **1** (20 µM ciano and 100 µM pink) and GPGKLVFF (20 µM purple and 100 µM green)

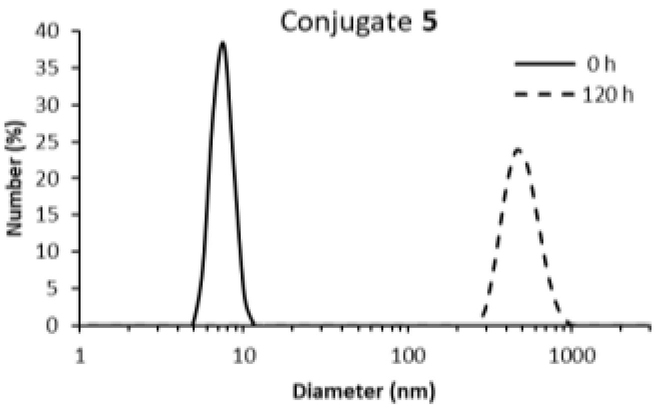

Figure S15. DLS size distributions by number % for conjugate **5** at t= 0 and after 120 h incubation at 37 °C in Phosphate buffer.

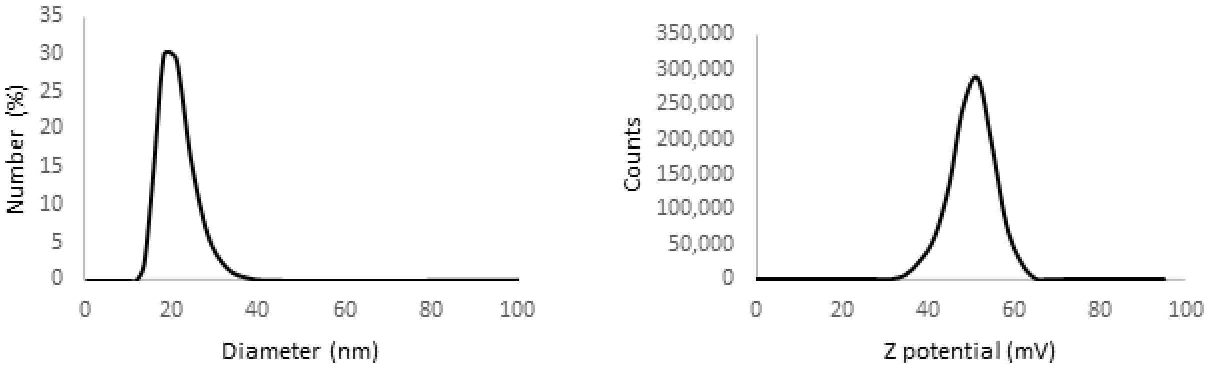

Figure S16. DLS size distribution by number % (left) and Electrophoretic Light Scattering (ELS) zeta potential (right) of compound **5** at 20 µM concentration.

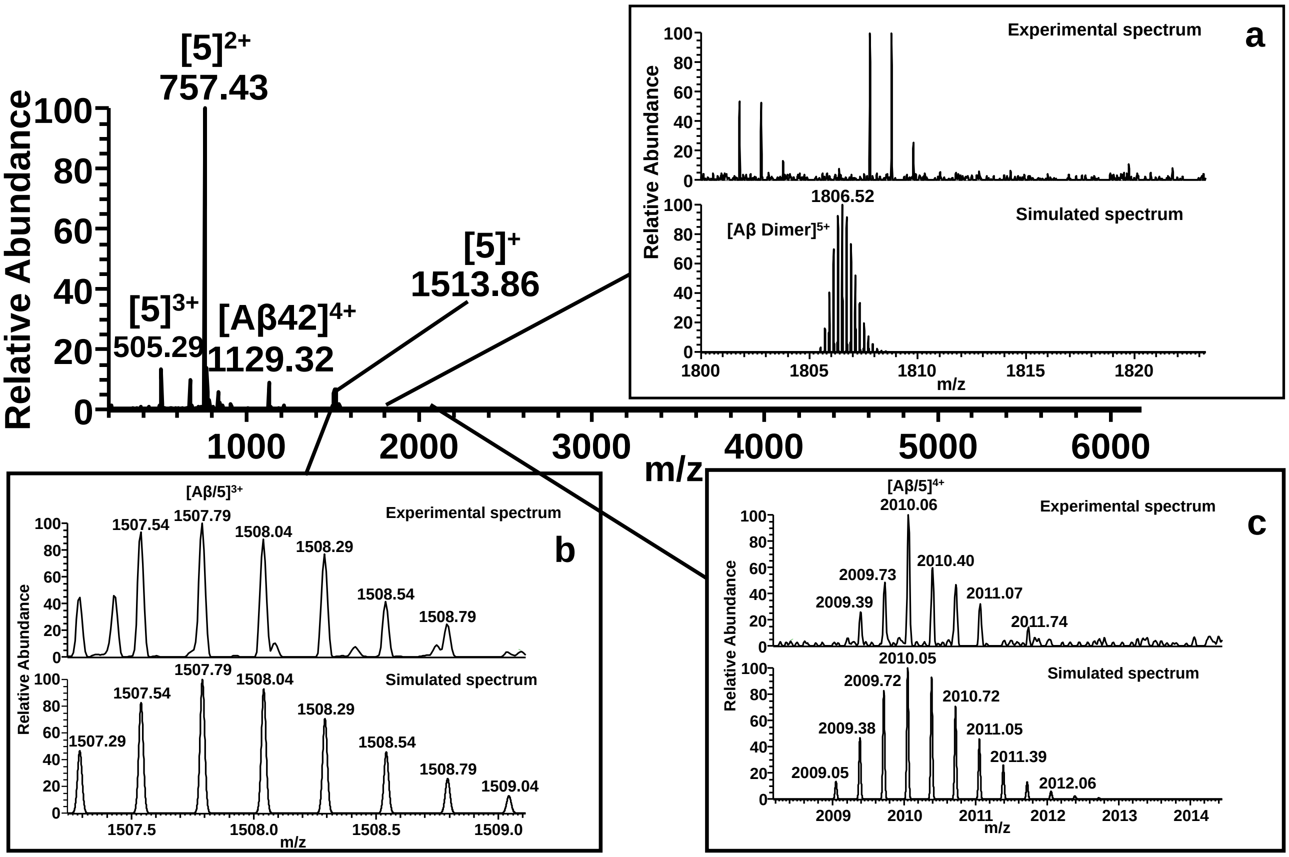

Figure S17. ESI-MS spectrum of $A\beta_{42}$/**5** sample. **Panel a** shows the comparison between the experimental and theoretical isotopic distribution of peak corresponding to the $A\beta_{42}$ dimer in the +5 charge state. **Panel b** and **c** show the comparison between the experimental and theoretical isotopic distribution of peaks corresponding to the $A\beta_{42}$/**5** adduct in the +3 and +4 charges state, respectively.

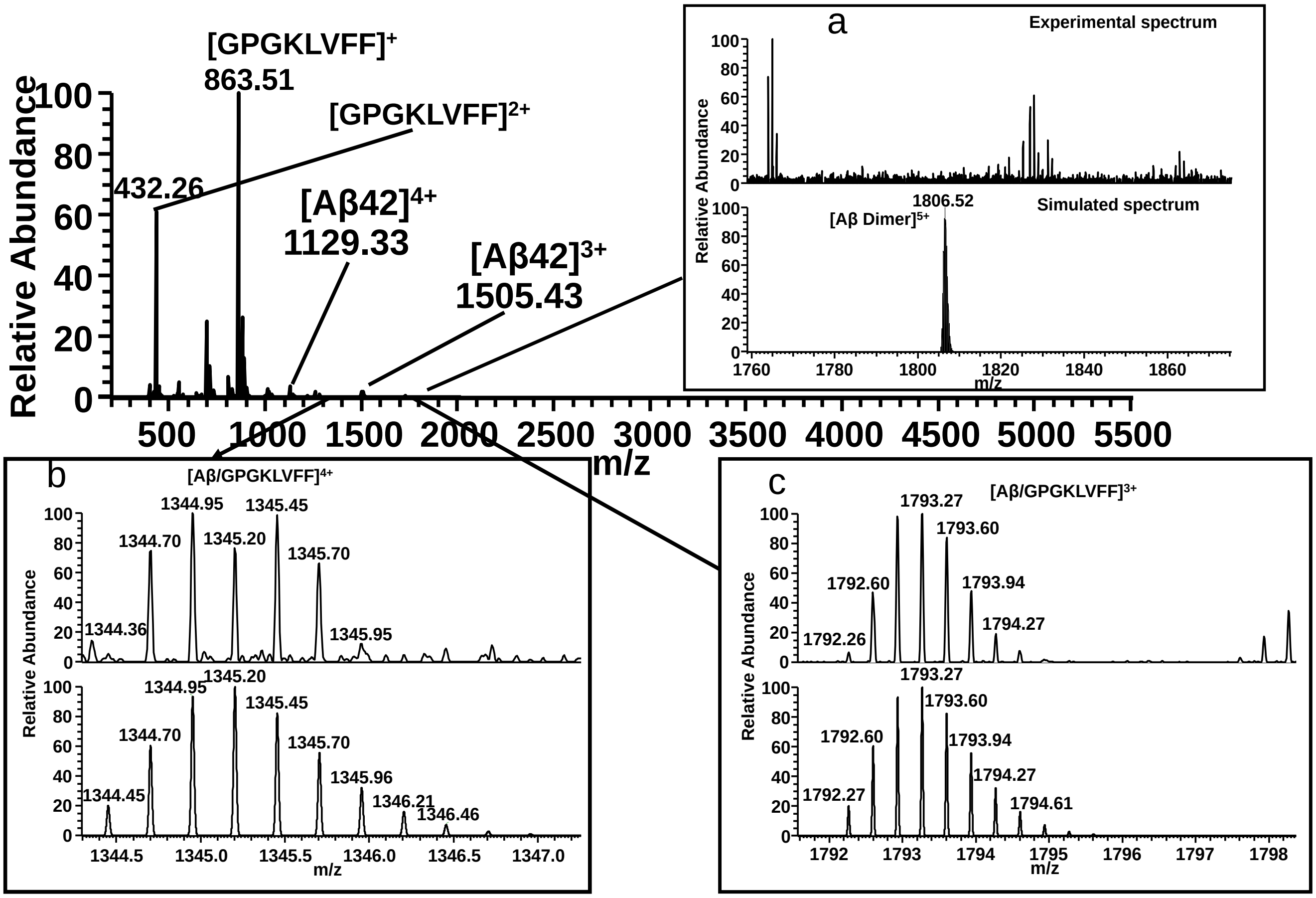

Figure S18. ESI-MS spectrum of Aβ_{42}/GPGKLVFF sample. **Panel a** shows the comparison between the experimental and theoretical isotopic distribution of peak corresponding to the Aβ_{42} dimer in the +5 charge state. **Panel b and c** show the comparison between the experimental and theoretical isotopic distribution of peak corresponding to the Aβ42/GPGKLVFF adduct in the +3 and +4 charges state, respectively.

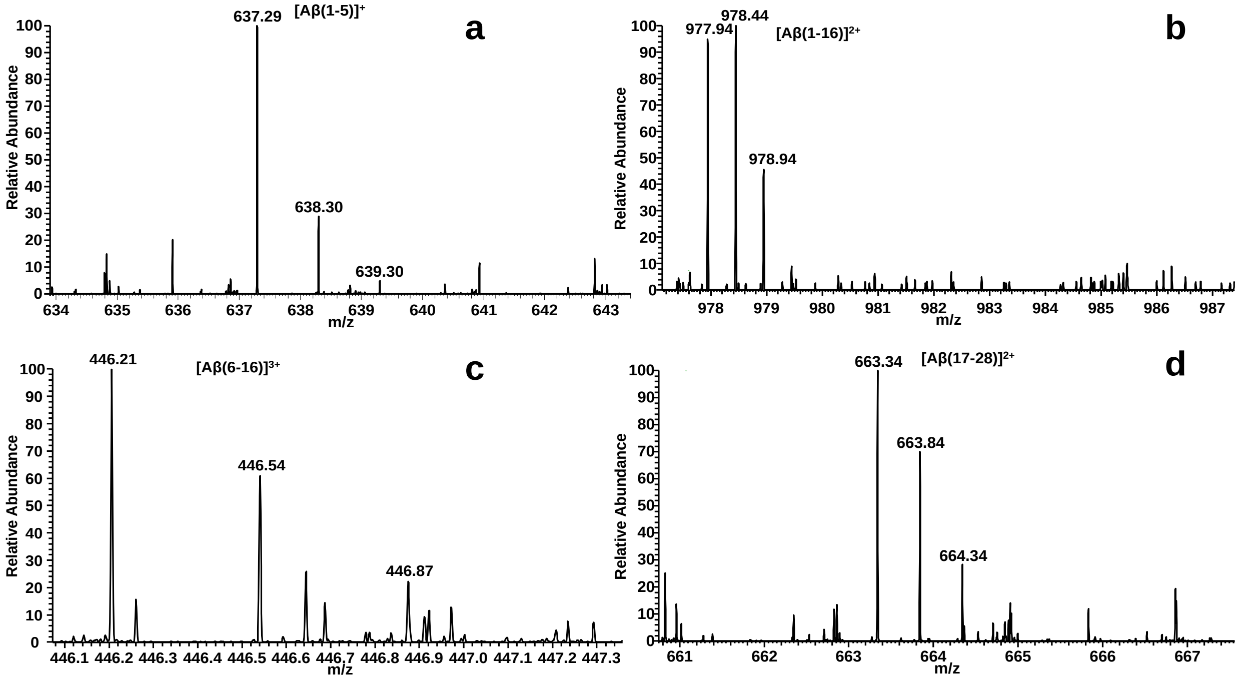

Figure S19. Isotopic patterns of peptide fragment produced after 2 h of Aβ$_{42}$ digestion with trypsin. **a)** Aβ(1-5), **b)** Aβ(1-16), **c)** Aβ(6-16) and **d)** Aβ(17-28).

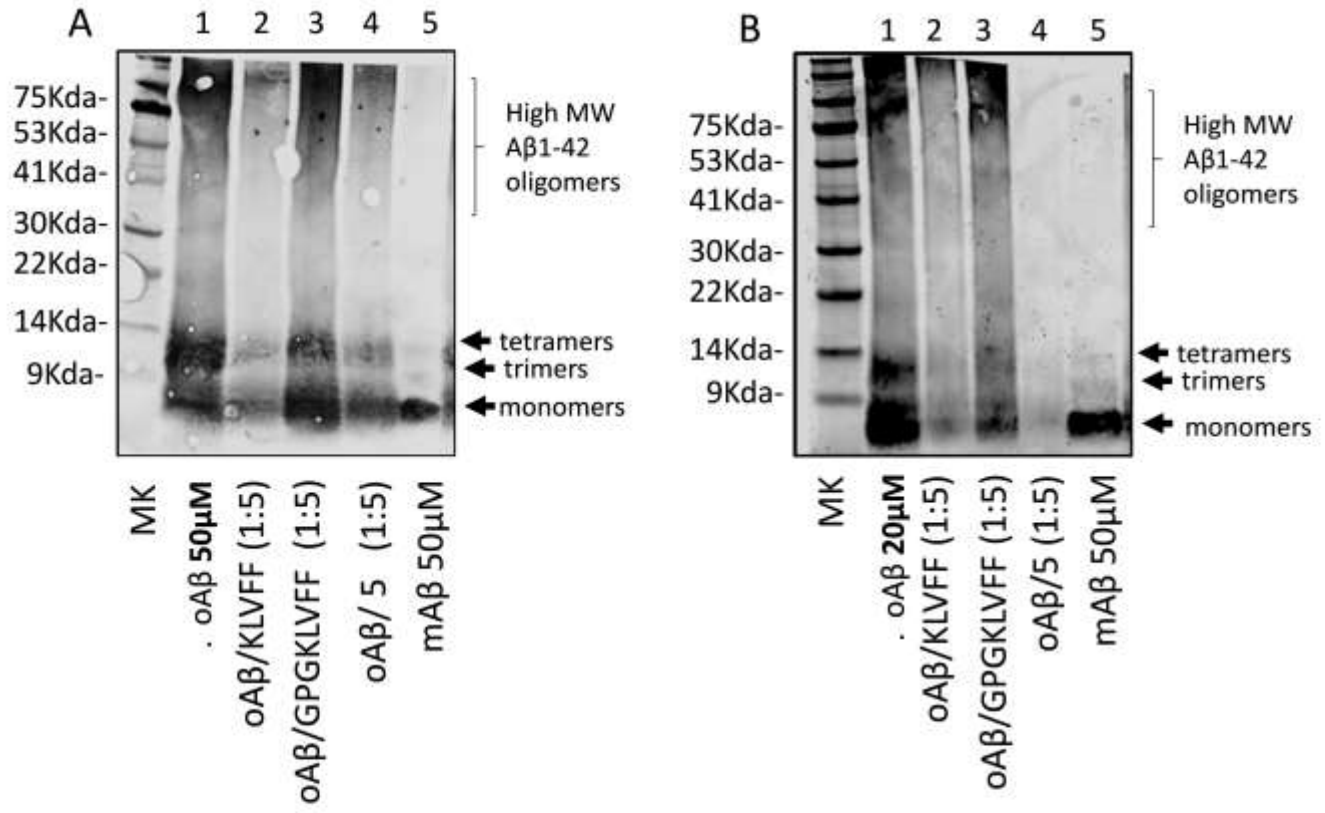

Figure S20. Representative western blot showing two different concentrations of Aβ oligomers obtained after 48h incubation at 4°C in the presence or absence of KLVFF peptide, GPGKLVFF and compound **5** at the Aβ/peptide ratio of 1:5. Samples were separated onto a 4–12% Bis·Tris SDS-PAGE gel and blotted with anti-Aβ N-terminal 1-16 mouse monoclonal antibody 6E10. Figures show that $A\beta_{42}$ gives rise to high-molecular-weight oligomeric species (lane 1), whose signal is strongly decreased when co- incubated with compound **5** (lane 4).
